# Transcriptome and DNA methylome divergence of inflorescence development between 2 ecotypes in *Panicum hallii*

**DOI:** 10.1093/plphys/kiad209

**Published:** 2023-04-05

**Authors:** Xiaoyu Weng, Haili Song, Avinash Sreedasyam, Taslima Haque, Li Zhang, Cindy Chen, Yuko Yoshinaga, Melissa Williams, Ronan C O’Malley, Jane Grimwood, Jeremy Schmutz, Thomas E Juenger

**Affiliations:** Department of Integrative Biology, University of Texas at Austin, TX 78712, USA; Department of Integrative Biology, University of Texas at Austin, TX 78712, USA; HudsonAlpha Institute for Biotechnology, Huntsville, AL 35806, USA; Department of Integrative Biology, University of Texas at Austin, TX 78712, USA; Department of Integrative Biology, University of Texas at Austin, TX 78712, USA; US Department of Energy Joint Genome Institute, Lawrence Berkeley National Laboratory, Berkeley, CA 94720, USA; US Department of Energy Joint Genome Institute, Lawrence Berkeley National Laboratory, Berkeley, CA 94720, USA; HudsonAlpha Institute for Biotechnology, Huntsville, AL 35806, USA; US Department of Energy Joint Genome Institute, Lawrence Berkeley National Laboratory, Berkeley, CA 94720, USA; HudsonAlpha Institute for Biotechnology, Huntsville, AL 35806, USA; HudsonAlpha Institute for Biotechnology, Huntsville, AL 35806, USA; Department of Integrative Biology, University of Texas at Austin, TX 78712, USA

## Abstract

The morphological diversity of the inflorescence determines flower and seed production, which is critical for plant adaptation. Hall's panicgrass (*Panicum hallii*, *P. hallii*) is a wild perennial grass that has been developed as a model to study perennial grass biology and adaptive evolution. Highly divergent inflorescences have evolved between the 2 major ecotypes in *P. hallii*, the upland ecotype (*P. hallii var hallii*, HAL2 genotype) with compact inflorescence and large seed and the lowland ecotype (*P. hallii var filipes*, FIL2 genotype) with an open inflorescence and small seed. Here we conducted a comparative analysis of the transcriptome and DNA methylome, an epigenetic mark that influences gene expression regulation, across different stages of inflorescence development using genomic references for each ecotype. Global transcriptome analysis of differentially expressed genes (DEGs) and co-expression modules underlying the inflorescence divergence revealed the potential role of cytokinin signaling in heterochronic changes. Comparing DNA methylome profiles revealed a remarkable level of differential DNA methylation associated with the evolution of *P. hallii* inflorescence. We found that a large proportion of differentially methylated regions (DMRs) were located in the flanking regulatory regions of genes. Intriguingly, we observed a substantial bias of CHH hypermethylation in the promoters of FIL2 genes. The integration of DEGs, DMRs, and *K_a_*/*K_s_* ratio results characterized the evolutionary features of DMR-associated DEGs that contribute to the divergence of the *P. hallii* inflorescence. This study provides insights into the transcriptome and epigenetic landscape of inflorescence divergence in *P. hallii* and a genomic resource for perennial grass biology.

## Teaser

A comparative transcriptome and DNA methylome analysis of different stages of inflorescence between upland and lowland ecotypes reveals gene expression and DNA methylation divergence in Panicum hallii.

## Introduction

Flowering plants have evolved diverse inflorescence architecture, which has a direct effect on the spatial arrangement of inflorescence branching and the production of flowers and seeds (Harder and Prusinkiewicz 2013; Kellogg 2022). The extensive diversity of inflorescence architecture is shaped by a combination of genetic, epigenetic, and environmental factors, with critical economic importance in agricultural crops and profound ecological implications in wild species (Barazesh and McSteen 2008; Teo et al. 2014; Tu et al. 2019). Recently, progress has been made in understanding the area of natural genetic architecture underlying inflorescence development, largely focusing on the model plants Arabidopsis (*Arabidopsis thaliana*) and several important crops, including rice (*Oryza sativa*), maize (*Zea mays*), common wheat (*Triticum aestivum*), and Setaria (*Setaria viridis*) (Kellogg et al. 2013; Zhang and Yuan 2014). This accumulated knowledge provides an opportunity to better understand the role of inflorescence diversity in the adaptive evolution of wild plants.

DNA methylation is a heritable epigenetic modification that contributes to gene regulation and genome structure and integrity (Chan et al. 2005; Law and Jacobsen 2010; Zhang et al. 2018). In land plants, DNA methylation occurs at the cytosine bases with 3 sequence contexts (CG, CHG, and CHH, where H represents A, T, or C). Genome-scale DNA methylation analyses show extensive variation among different plant species in all 3 DNA methylation contexts, with the predominant form being CG methylation compared with CHG and CHH methylation (Niederhuth et al. 2016). The classic model assumes that the addition of DNA methylation in the promoters of genes typically represses gene expression by recruiting repressor proteins (Tate and Bird 1993). Recently, a growing body of research has revealed that gene body methylation can be positively associated with gene expression and may shape important features of plant genome evolution (Bewick and Schmitz 2017). DNA methylation plays an essential role in a wide range of growth and development events, especially in the developmental complexity of inflorescence architecture (Zhang et al. 2018; Tu et al. 2019). This perspective is supported by evidence that most loss-of-function mutations of genes involved in DNA methylation establishment and maintenance show abnormal inflorescence morphology (Moritoh et al. 2012; Fernandez-Nohales et al. 2014; Liao et al. 2019). Additionally, epigenetic alleles involving DNA methylation variation have been identified as the key regulators of inflorescence development (Zhu et al. 2013; Zhang et al. 2017; Xu et al. 2020). These findings suggest that DNA methylation may be of crucial importance in the evolution of the structure and organization of the inflorescence.

High-throughput sequencing techniques have been used extensively for genome-wide profiling of gene expression and DNA methylation to study a variety of developmental processes (Yang et al. 2015; Huang et al. 2019; Rajkumar et al. 2020; Shi et al. 2021). As the key determinant of productivity, the inflorescence of many crop species has been studied with detailed developmental stage-specific transcriptome profiling (Furutani et al. 2006; Wang et al. 2010; Eveland et al. 2014; Harrop et al. 2016; Feng et al. 2017; Zhu et al. 2018). For example, stage- and meristem-specific gene expression profiles have provided a genome-wide view of regulatory networks controlling young panicle development in rice (Furutani et al. 2006; Wang et al. 2010; Harrop et al. 2016) and wheat (Feng et al. 2017). Moreover, whole-genome analysis of DNA methylation has found epigenetic mechanisms that coordinate gene structure and expression during inflorescence development (Li et al. 2012; Parvathaneni et al. 2020; Sun et al. 2020). For instance, single-base resolution methylome studies have assessed the functional importance of epigenetic differentiation of young panicles between wild and cultivated rice (Li et al. 2012). Genome-wide DNA methylation profiling integrated with other multi-omics analysis has revealed the role of chromatin interactions that coordinate *trans* and *cis* regulation of differential expression between 2 separate types of inflorescences (ear and tassel) in maize (Sun et al. 2020). These advances provide not only a deep understanding of the relationship between complex gene regulatory networks and epigenetic modifications but also help to identify the potential candidates controlling inflorescence morphology and grain yield.

Hall's panicgrass (*Panicum hallii*, *P. hallii*) is a native perennial C_4_ grass with a distribution in southwestern regions of North America (Lowry et al. 2015). Due to a close evolutionary relationship to the polyploid biofuel crop switchgrass (*Panicum virgatum*), *P. hallii* has been developed as a complementary diploid model system (Lovell et al. 2018). *P. hallii* is found in a wide range of soil and ecological conditions, spanning from xeric inland regions to mesic coastal areas (Gould et al. 2018; Palacio-Mejia et al. 2021). *P. hallii* populations have diverged into 2 major ecotypes (or varieties), *P. hallii var. hallii* (hereafter *var. hallii*) and *P. hallii var. filipes* (hereafter *var. filipes*) (Lowry et al. 2015; Lovell et al. 2018). Similar to other upland plants, the widespread *var. hallii* is typically found in drier habitats with shallow and rocky soils (Palacio-Mejia et al. 2021). In contrast, the more geographically restricted *var. filipes* commonly grows in Gulf Coast areas in clay soils and mesic depressions (Palacio-Mejia et al. 2021). Whole-genome sequencing and assemblies suggest that *var hallii* and *var filipes* shared a common ancestor ∼1.08 million years ago (Mya) (Lovell et al. 2018). Although there is some evidence of hybridization between these ecotypes, it is rare in nature and they exhibit considerable population structure and genomic and phenotypic divergence, including notable differences in flowering time, plant size, and inflorescence architecture (Palacio-Mejia et al. 2021). In general, *var. hallii* flowers earlier than *var. filipes* and is distinguished from the latter by its sparse inflorescence and larger seed. Recently, genetic resources derived from the crossing of *var. hallii* with *var. filipes* have been developed for studying the genetic basis of ecotype-differentiating traits (e.g. flowering time, flower number, seed mass, etc.), shoot–root resource acquisition traits, and seed dormancy and seedling characteristics (Lowry et al. 2015; Khasanova et al. 2019; Razzaque and Juenger 2022). Transcriptome studies have been undertaken with the goal of understanding how *P. hallii* responds to various environmental cues (Lovell et al. 2016; Weng et al. 2019). Nevertheless, gene expression divergence associated with the evolution of ecotype-specific morphology and its relationship with the global patterns of DNA methylation variation remain poorly understood in *P. hallii*.

In this study, we performed a comparative transcriptome and DNA methylome analysis at different stages of inflorescence development contrasting the 2 ecotypes of *P. hallii* using RNA sequencing and whole-genome bisulfite sequencing. Global analysis of transcriptome data identified the heterochronic patterns of differentially expressed genes (DEGs) between the 2 types of *P. hallii* inflorescences over development. Similarly, comparing whole-genome DNA methylation profiles allowed a characterization of DNA methylation divergence during the evolution and development of *P. hallii* inflorescence. An integrated analysis of differentially methylated regions (DMRs), DEGs, and *K_a_*/*K_s_* ratio highlighted the evolutionary features of candidate genes that might determine the phenotypic diversity of inflorescence branching architecture and seed size in *P. hallii*. Together, this study provides insights into the transcriptome and epigenetic landscape of inflorescence divergence in *P. hallii*.

## Results

### Distinct phenotypes of inflorescence and seed between 2 *P. hallii* ecotypes

**Figure 1.**
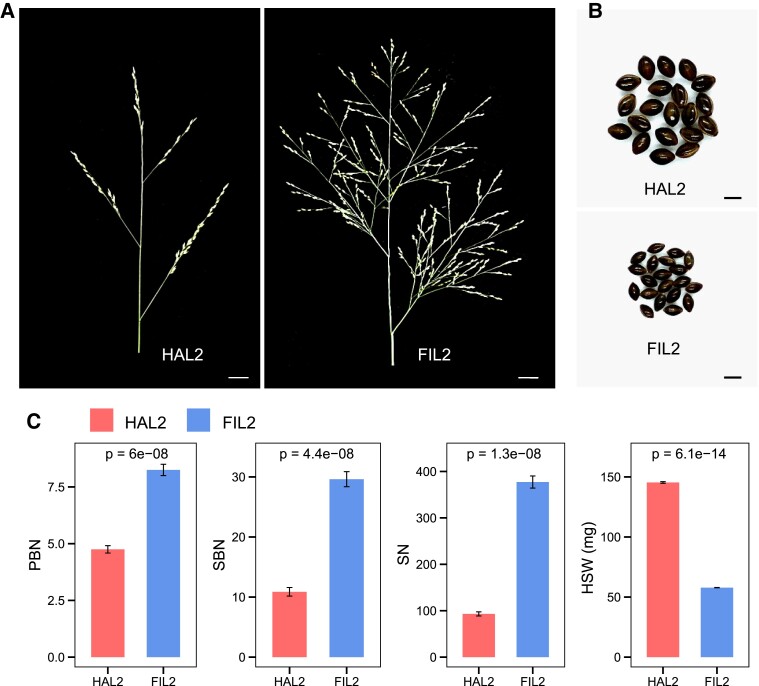
Morphological differences between 2 representatives of the upland (HAL2) and lowland (FIL2) ecotypes in *P. hallii*. Representative image of the inflorescence **(A)** and seed **(B)** morphology of HAL2 and FIL2 (scale bar in **A**, 1 cm; scale bar in **B**, 1 mm). **C)** Primary branch numbers (PBN), secondary branch numbers (SBN), spikelet numbers (SN), and hundred-seed weight (HSW, mg) in HAL2 and FIL2 plants. In all panels, the bars and error bars are the average values and *SE*, respectively, based on the measurements from 8 replicates. The *P*-values were determined by Student's *t*-test.

To provide tools for studying *P. hallii* evolutionary genomics, we have developed reference genomes spanning the wide ecotypic divergence observed in *P. hallii* (Lovell et al. 2018). Our genome assemblies have been derived from 2 accessions, *P. hallii var. hallii* (HAL2) and *P. hallii var. filipes* (FIL2), that are representative of the upland and lowland ecotypes in *P. hallii* (Lovell et al. 2018). In this study, we investigate inflorescence development and divergence between HAL2 and FIL2. As shown in Fig. 1A, *P. hallii* has a panicle-type inflorescence with many branches supporting spikelet development and seed set. The inflorescence of HAL2 exhibits remarkably different branching patterns compared with that in FIL2, mainly in the reduction of both primary and secondary branch numbers (Fig. 1, A and C), and its compact rather than open structure. This divergent architecture results in a significant decrease in spikelet numbers in HAL2 (Fig. 1C). In contrast, we observed significantly enlarged seed size in HAL2 relative to FIL2, as measured by 100-seed weight (Fig. 1, B and C). These observations suggested that divergence in inflorescence architecture in *P. hallii* may be associated with a trade-off between seed size and number in *P. hallii* as has been observed in many domesticated grasses (Sadras 2007). To determine the developmental origin of the differences, we performed scanning electron microscope (SEM) experiments to compare the inflorescence between HAL2 and FIL2 at the early stages (D1 and D2, see the “Plant materials and sample collection” and “Scanning electron microscope” sections in method for details). We observed a strong gradient of development at the D1 stage, which included both later branching meristems and floral meristems (Supplemental Fig. S1). While D2 stage is mainly spikelet meristems and floral meristems, it still has branching meristems at the base (Supplemental Fig. S1). SEM imagery showed that the number of branching meristems was substantially higher in FIL2 compared with HAL2 (Supplemental Fig. S1), which likely explains the morphological difference in inflorescence between HAL2 and FIL2.

### Genome-wide analysis of gene expression divergence between 2 *P. hallii* inflorescences

**Figure 2.**
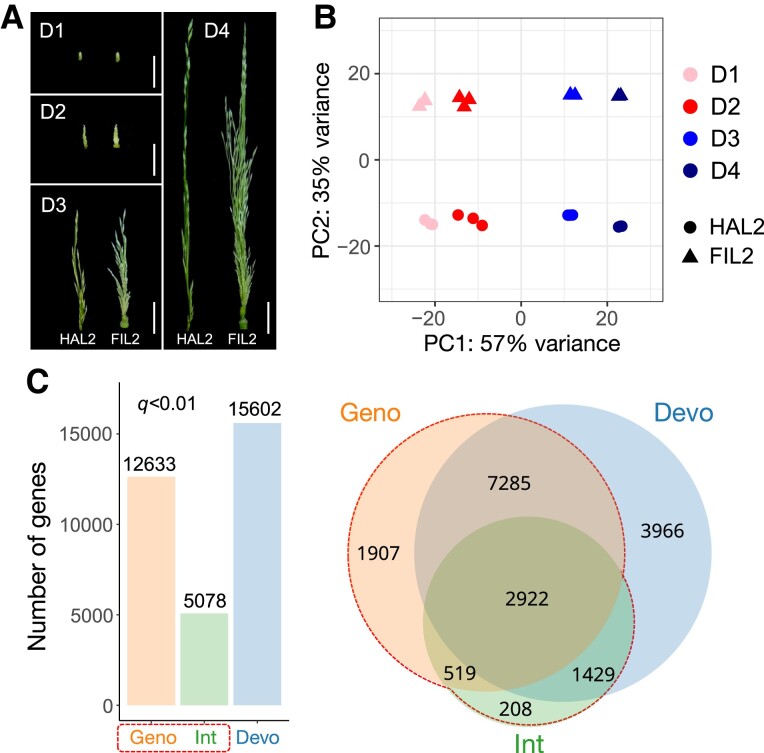
Global transcriptome analysis of gene expression divergence between HAL2 and FIL2 inflorescence. **A)** Four stages of inflorescence tissues from HAL2 and FIL2 were collected for RNA-seq. Different stages were designated as D1–D4 according to the lengths of young inflorescences (scale bar, 1 cm) (see method for details). **B)** Principal component analysis of the RNA-seq data for the 24 inflorescence samples showing the developmental signatures and genotype effects. **C)** Bar plot and Venn diagrams depict genes that are differentially regulated with genotype effects (Geno), development effects (Devo), and genotype-by-development interaction effects (Int) in factorial linear modeling (*q* < 0.01). DEGs with significant genotype and/or interaction effects were defined as genes with divergent expression between HAL2 and FIL2 inflorescence.

To explore this divergent inflorescence development, RNA-seq experiments were performed on 4 stages of inflorescence tissues, designated as D1–D4 of HAL2 and FIL2 (2 genotypes × 4 developmental stages × 3 biological replicates = 24 libraries) (Fig. 2A, see method for details). After filtering genes with low expression, 19,332 one-to-one orthologous genes were detected in the dataset for downstream analysis. There were strong correlations among the biological replicates (*r* > 0.97), supporting the high quality and reproducibility of the entire dataset. Principal component analysis of expressed genes revealed a strong global structure along the development gradient and related to genotype divergence (Fig. 2B). The first component explained 57% of the expression variance and clearly distinguished the stages across the developmental gradient, while the second component explained 35% of the expression variance and mainly discriminated between samples from HAL2 and FIL2 (Fig. 2B). The first 2 components explained the vast majority of variance (92%), suggesting the dominance of development and genotype effects in the entire dataset.

To analyze the molecular basis of expression divergence, we applied linear models on gene counts to test the effects of genotype, development, and genotype × development interaction on gene expression across the entire transcriptome (see method for details). We identified 12,633 genes (65.3%) with significant genotype effects (*q_geno_* < 0.01) and 15,602 genes (80.7%) with significant expression level changes across the developmental gradient (*q_devo_* < 0.01) (Fig. 2C and Supplemental Table S1). Meanwhile, we detected 5,078 genes (26.3%) with significant interaction between development and genotype (*q_int_* < 0.01) (Fig. 2C and Supplemental Table S1), with the magnitude or direction of gene expression divergence between HAL2 and FIL2 depended on the specific stage of development. We only detected 1,907 genes (9.9%) with strictly additive genotype effects, which are genes with consistent difference between HAL2 and FIL2 regardless of developmental stages (Fig. 2C and Supplemental Table S1). Similarly, we detected 3,966 genes (20.5%) exhibiting strictly additive developmental effects without genotype influences (genotype and/or interaction effects) (Fig. 2C and Supplemental Table S1). Finally, we found that 7,285 genes (37.7%) were detected with independent genotype and development effects, but without significant interaction effects (Fig. 2C and Supplemental Table S1). After examining genes exhibiting significant genotype and/or interaction effects (*q* < 0.01), we concluded that 14,270 genes (73.8%) showed expression divergence between HAL2 and FIL2 inflorescence (Fig. 2C and Supplemental Table S1).

### Heterochronic changes in gene expression divergence during inflorescence development

**Figure 3.**
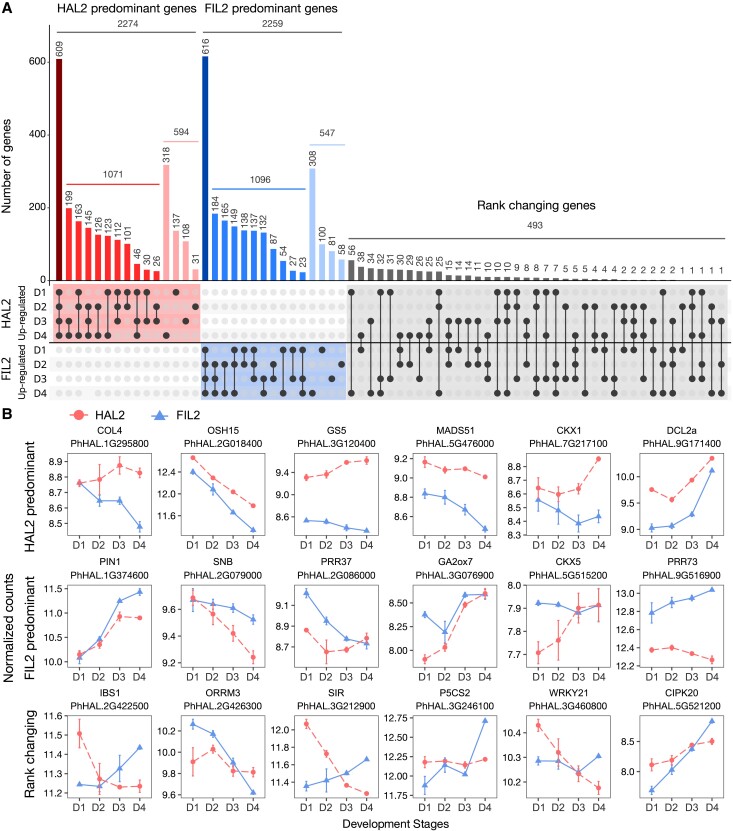
Stage-specific contrast of interaction genes during inflorescence development. **A)** Quantification of stage-specific expression of interaction genes as HAL2 predominant, FIL2 predominant, and rank changing patterns. The numbers of genes showing developmental-specific expression patterns in 1 or more the sampling stages are shown in vertical bars of the figure. Dots at the bottom of each vertical bar indicate the developmental-specific expression identified at each sampling stage. The lined dots indicate 2 or more sampling stages showing differential expression between 2 genotypes. **B)** Expression of interaction genes with the HAL2 predominant (upper), FIL2 predominant (middle), and rank changing patterns (bottom). The *x*-axis represents 4 developmental stages, while the *y*-axis represents normalized counts using variance stabilizing transformation in DEseq2. In all panels, the points and error bars are the average values and *SE*, respectively, based on normalized counts of 3 RNA-seq replicates. The gene ID and the names of their putative orthologs are shown on the top of the expression pattern plots.

Our SEM results revealed heterochrony of inflorescence development, a divergence in the timing of development between the 2 ecotypes. Thus, we primarily focused on 5,078 interaction genes that could be responsible for this phenomenon. We conducted a stage-by-stage contrast of genes with diverged expression between HAL2 and FIL2 inflorescence to determine the direction of differential expression (*q* < 0.01, Supplemental Table S2). We found that the vast majority of interaction genes (4,533, 89.3%) showed patterns with consistently greater expression in one of the ecotypes (Fig. 3A); for convenience, we call these HAL2 or FIL2 predominant expression patterns. We did not observe a directional bias in the pattern of predominant genes, since 2,274 genes showed HAL2 predominant patterns and 2,259 genes exhibited FIL2 predominant patterns (Fig. 3A). For example, we identified putative orthologs controlling flowering time (*CONSTANS-LIKE 4* [*COL4*], *MADS-BOX TRANSCRIPTION FACTOR 51* [*MADS51*], *PSEUDO-RESPONSE REGULATOR 37* and *73* [*PRR37* and *PRR73*]), organ development (*ORYZA SATIVA HOMEOBOX 15* [*OSH15*], *GRAIN SIZE 5* [*GS5*], *SUPERNUMERARY BRACT* [*SNB*]), hormone pathways (*CYTOKININ DEHYDROGENASE 1* and *5* [*CKX1* and *CKX5*], *PIN-FORMED 1* [*PIN1*], *GIBBERELLIN 2-OXIDASE 7* [*GA2ox7*]), and small RNA biogenesis (*DICER-LIKE PROTEIN 2A* [*DCL2a*]) among these genes (Fig. 3B). These candidates could be either HAL2 or FIL2 predominant expression patterns (Fig. 3B), which have pleiotropic effects in inflorescence development in many grass systems (Bouche et al. 2006; Lee et al. 2007; Barazesh and McSteen 2008; Yan et al. 2013). We found 493 genes (9.7%) with rank changing patterns of relative repression or induction changes between genotypes at different development stages (Fig. 3A). These genes had either an opposite direction or a remarkable magnitude difference in gene expression divergence. Many of them were associated with multiple stress pathways, including putative orthologs of *IMPAIRED IN BABA-INDUCED STERILITY 1* (*IBS1*), *ORGANELLE RNA RECOGNITION MOTIF-CONTAINING 3* (*ORRM3*), *SULFITE REDUCTASE* (*SIR*), *PYRROLINE-5-CARBOXYLATE SYNTHETASE 2* (*P5CS2*), *WRKY DNA-BINDING PROTEIN 21* (*WRKY21*), and *CBL-INTERACTING PROTEIN KINASE 20* (*CIPK20*) (Fig. 3B).

**Figure 4.**
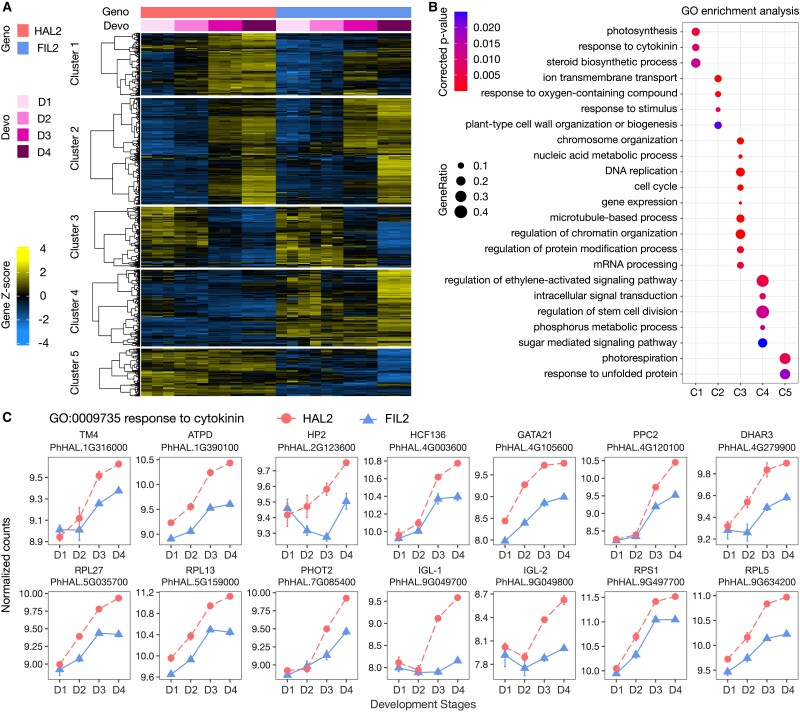
Divergence patterns of interaction genes during inflorescence development. **A)** Heatmaps of gene expression with interaction genes between HAL2 and FIL2 across 4 developmental stages. Only gene expression data from 5,078 interaction genes are used for clustering. The minimum centroid distance was used to determine the number of cluster cores. The genotype and development information is added on top as color bars. **B)** The dot plot of the most significantly enriched GO terms from each cluster (*y*-axis) in 5,078 interaction genes. The size of the dots represents the number of genes in the significant DEG list associated with the GO term and the color of the dots represents the FDR corrected *P*-value (Benjamini–Hochberg method). **C)** Expression of genes from the enriched GO term of “response to cytokinin” (GO:0009735). The *x*-axis represents 4 developmental stages, while the *y*-axis represents normalized counts using variance stabilizing transformation in DEseq2. In all panels, the points and error bars are the average values and *SE*, respectively, based on normalized counts of 3 RNA-seq replicates. The gene ID and the names of their putative orthologs are shown on the top of the expression pattern plots.

To further gain insight into the divergence patterns across developmental gradients, we conducted a clustering analysis for the 5,078 interaction genes. The minimum centroid distance was used to determine the number of cluster cores (*c*) (Supplemental Fig. S2). This analysis led to the detection of 5 core clusters representing the divergence pattern of interaction gene expression across development, ranging from 681 to 1,528 genes in each cluster (Fig. 4A and Supplemental Table S3). The expression of genes in Clusters 1 and 2 had an increasing tendency across developmental gradients (Fig. 4A). By contrast, genes in Cluster 3 had expression that gradually decreased with the maturation of the inflorescence (Fig. 4A). The majority of genes in Clusters 4 and 5 displayed FIL2 and HAL2 predominant patterns, respectively (Fig. 4A). We next performed Gene Ontology (GO) enrichment analysis for each cluster, in which the significance was determined by the False Discovery Rate (FDR) corrected *P*-value <0.05. This analysis identified GO terms that were significantly enriched in each cluster, e.g. photosynthesis and response to cytokinin terms in Cluster 1; ion transmembrane transport and multiple responses to stress terms in Cluster 2; chromosome organization, DNA replication, cell cycle, gene expression, and mRNA processing terms in Cluster 3; regulation of ethylene-activated signaling pathway and intracellular signal transduction terms in Cluster 4; and photorespiration term in Cluster 5 (top terms in Fig. 4B, the full list in Supplemental Table S4). We were particularly interested in the expression patterns of genes in an enriched GO term of “response to cytokinin” (GO:0009735) (FDR corrected *P* = 0.0086) in Cluster 1, as cytokinin is often a key factor in determining the architecture of the inflorescence. The genes in this enriched term were putative orthologs of *GATA TRANSCRIPTION FACTOR 21* (*GATA21*), *HISTIDINE-CONTAINING PHOSPHOTRANSFER 2* (*HP2*), ribosomal protein (*RIBOSOMAL PROTEIN UL5C* [*RPL5*], *RIBOSOMAL PROTEIN UL13C* [*RPL13*], *RIBOSOMAL PROTEIN BL27C* [*RPL27*], and *RIBOSOMAL PROTEIN S1* [*RPS1*]), and other genes involved in the modulation of cytokinin homeostasis (Fig. 4C). Intriguingly, the increasing trend across developmental gradients of these genes in HAL2 is much stronger than that in FIL2 (Fig. 4C), suggesting that heterochronic changes in the timing of cytokinin signaling could be a crucial driver in the divergence of HAL2 and FIL2 inflorescence development.

We also note that there is a large number of genes (7,285) with independent genotype and development effects. The directions and distribution of DEGs in this group are depicted in Supplemental Fig. S3. After conducting a clustering analysis of this gene set, we identified 6 core clusters with different patterns of gene expression behavior (Supplemental Figs. S2 and S4A). Among them, genes in Clusters 1 and 2 had an increasing tendency across developmental gradients, while genes in Clusters 3 and 4 decreased the expression with the maturation of the inflorescence (Supplemental Fig. S4A). The majority of genes in Clusters 5 and 6 displayed FIL2 and HAL2 predominant patterns, respectively (Supplemental Fig. S4A). We identified an enriched GO term of “response of auxin” (GO:0009733) in Cluster 1, which included putative orthologs of *INDOLE-3-ACETIC ACID INDUCIBLE 4* and *31* (*IAA4* and *IAA31*) and *MYB DOMAIN PROTEIN 12*, *94*, and *96* (*MYB12*, *MYB94*, and *MYB96*) (Supplemental Fig. S4, B and C; Supplemental Table S4). We observed a considerable number of significantly enriched GO terms in Clusters 3 and 4, many of which had a shared function in metabolic processes, gene expression, and DNA repair (Supplemental Fig. S4B and Supplemental Table S4). Intriguingly, we detected enriched GO terms of “maintenance of inflorescence meristem identity” (GO:0010077) and “flower development” (GO:0009908) in Cluster 4, which included the putative orthologs of *BEL1-LIKE HOMEODOMAIN 8* and *9* (*BLH8* and *BLH9*) and *MADS-BOX TRANSCRIPTION FACTOR 15*, *17*, and *58* (*MADS15*, *MADS17*, *MADS58*) (Supplemental Fig. S4, B and C and Supplemental Table S4). Moreover, we found an enriched GO term of “methylation” (GO:0032259) in Cluster 4, which included the putative orthologs involved in epigenetic silencing and de novo methylation (*PROTEIN ARGININE METHYLTRANSFERASE 5* [*PRMT5*], *RNA-DIRECTED DNA METHYLATION 12* [*RDM12*], *METHYLTRANSFERASE 1* [*MET1*], and *EMBRYONIC FLOWER 2* [*EMF2*]) (Supplemental Fig. S4, B and C; Supplemental Table S4). Although not identified as interaction genes, these genes may still contribute to the expression divergence between HAL2 and FIL2 inflorescence. Finally, we found enriched GO terms of photosynthesis in Cluster 2, macromolecule modification in Cluster 5, and plastid organization in Cluster 6 (Supplemental Fig. S4B and Supplemental Table S4).

For 1,907 genes with strictly additive genotype effects, we identified 2 core clusters with different predominant patterns (Supplemental Figs. S2 and S5A). Enriched GO terms were not identified in these clusters; however, we observed the putative orthologs involved in flowering time (*EARLY HEADING DATE 3* [*Ehd3*]) and GA signaling (*GIBBERELLIC ACID INSENSITIVE* [*GAI*]) pathways with HAL2 predominant patterns (Cluster 1) and putative orthologs involved in DNA methylation (*METHYL-CPG-BINDING DOMAIN 10* [*MBD10*]) and early flower development (*MADS-BOX TRANSCRIPTION FACTOR 3* [*MADS3*]) pathways with FIL2 predominant patterns (Cluster 2) (Supplemental Fig. S5B). For 3,966 genes with strictly additive development effects, we identified 4 core clusters that were considered co-expressed (Supplemental Figs. S2 and S6A). We identified a large number of GO terms that were significantly enriched in Cluster 1, including the terms of metabolic processes, chromosome organization, gene expression, RNA splicing, cell cycle, and DNA recombination (Supplemental Fig. S6B and Supplemental Table S4). We observed a decreasing tendency of putative orthologs of *LONELY GUY* (*LOG*) and *RICE FLORICULA/LEAFY* (*RFL*) in Cluster 1 (Supplemental Fig. S6C), which are associated with meristem activity and initiation (Kurakawa et al. 2007; Rao et al. 2008). Finally, we identified enriched GO terms of vesicle transport in Cluster 2 and cell wall biogenesis in Cluster 3 (Supplemental Fig. S6B and Supplemental Table S4). As development progressed, the expression of genes in these terms (e.g. GO:0009834, plant-type secondary cell wall biogenesis) increased gradually, with no expression difference between the 2 ecotypes (Supplemental Fig. S6C).

### Global methylome profiles of different *P. hallii* inflorescences

As methylation-related genes and enriched GO terms were identified in the divergence expression analysis, we generated single-base resolution maps of DNA methylation using bisulfite sequencing to explore the possible function of DNA methylation in *P. hallii* inflorescence divergence. We utilized the same paired inflorescence tissues from HAL2 and FIL2 at the early (D1) and late (D4) stages from our RNA-seq studies for DNA methylation analysis (2 genotypes × 2 developmental stages × 3 biological replicates = 12 libraries). After removal of adapter contaminates and low-quality reads, a total of ∼1.9 billion paired-end reads were generated across our samples. We observed a strong mapping bias by performing alignments of the same sequencing reads from all inflorescence samples to both the HAL2 and FIL2 reference genomes. Mapping efficiencies dramatically dropped from ∼75% when aligned to “self” genomes to ∼30% when aligned to incorrect genomes (Supplemental Fig. S7 and Supplemental Table S5), demonstrating substantial sequence divergence between HAL2 and FIL2, especially in noncoding regions. Therefore, we mapped reads from each genotype to their respective genomes for further analysis. We found that ∼73% of CG sites, 68% of CHG sites, and 55% of CHH sites were covered by at least 5 uniquely mapped reads across different genotypes and tissues (Supplemental Fig. S8). We observed high bisulfite conversion rates, with an average level of 97.5% using a chloroplast control (Supplemental Table S5), that there was little strand differentiation, and the 3 biological replicates of each sample were highly correlated with each other (*r* > 0.95). These results suggested that our data were reproducible and sufficient for further analysis.

**Figure 5.**
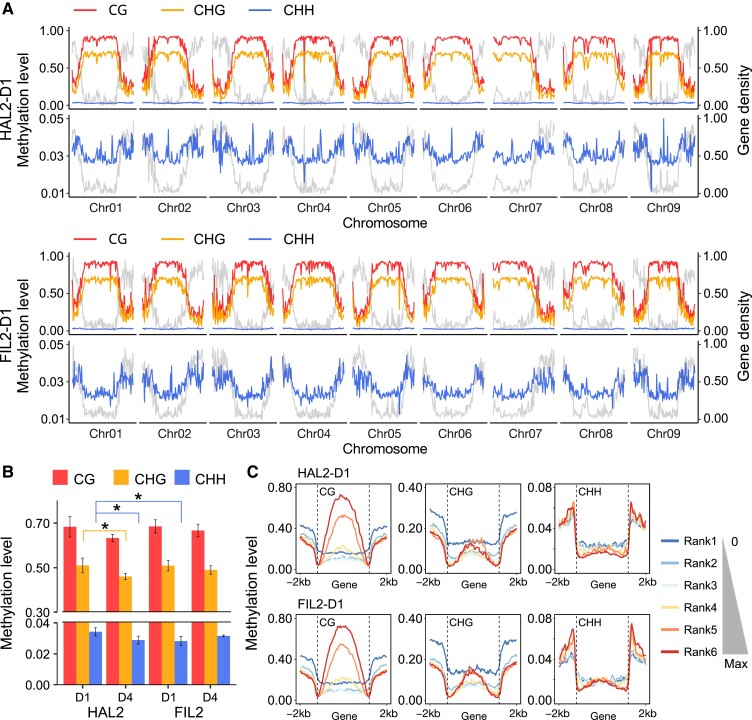
Global DNA methylation profiling and influence of DNA methylation on gene expression during inflorescence development. **A)** The distribution of CG, CHG, and CHH methylation levels (mean values of 3 biological replicates) and gene density across the HAL2 and FIL2 chromosomes from D1 inflorescence. **B)** DNA methylation levels in different inflorescence tissues and genotypes. The bars and error bars are the average values and *SE*. *P*-values less than 0.05 are labeled as asterisks (Student's *t*-test). **C)** Methylation level within gene body and 2 kb flanking regions in CG, CHG, and CHH contexts for the gene sets that are expressed at different levels in HAL2 and FIL2 from D1 inflorescence. The average of 3 replicates was displayed for CG, CHG, and CHH contexts. The data for D4 stage of inflorescences are given in Supplemental Figs. S10 and S12.

Genome-wide DNA methylation level analyses revealed that a large proportion of CG (∼66%) and CHG (∼49%) sites have methylated cytosines, while the level of CHH methylation (∼3.1%) was comparatively low (Fig. 5B). The genome-wide degree of CG methylation was stable across genotypes and developmental stages; however, we observed a significant difference in non-CG methylation levels (especially in CHH methylation) between genotypes or development stages (*P* < 0.05) (Fig. 5B). Global methylation levels revealed that around 32% of CG and 40% of CHG sites had low methylation levels (<0.2), and about 64% of CG and 32% of CHG sites showed high methylation levels (>0.8), while CHH site levels were overall very low, with about 97% in the low methylation level category (<0.2) and less than 0.2% had high methylation levels (>0.8) (Supplemental Fig. S9). The distributions of methylation levels were further compared in 3 contexts across chromosomes (Fig. 5A and Supplemental Fig. S10). We observed a broad hyper CG and CHG methylation region for each chromosome, which is highly negatively correlated with gene density (*r* = −0.974 to −0.976 for CG in all samples; *r* = −0.975 to −0.976 for CHG in all samples) (Fig. 5A and Supplemental Fig. S10). These regions are clearly associated with pericentromeres, which have been identified in recent *P. hallii* genomic studies (Lovell et al. 2018). We found a strong positive correlation between CHH methylation levels and gene density in most samples (*r* = 0.573 in HAL2-D1, *r* = 0.741 in FIL2 D1, *r* = 0.626 in FIL2-D4) (Fig. 5A). Intriguingly, this positive correlation was relatively weak in HAL2 inflorescence at the late stage (*r* = 0.199 in HAL2-D4) (Supplemental Fig. S10).

To understand the relationship between DNA methylation and gene expression, we profiled DNA methylation levels across gene bodies for genes with different expression levels. Genes were divided into 6 groups based on expression, from a silent rank1 (count = 0) to the highest rank6 (Supplemental Fig. S11). We observed that genes with low expression, especially the genes with no expression in rank1, have higher CG and CHG methylation levels at the promoter and the 3′ end regulatory regions (Fig. 5C and Supplemental Fig. S12). Notably, we found that genes with high expression, especially the genes in rank6 and rank5, have higher CG methylation levels at the gene body regions (Fig. 5C and Supplemental Fig. S12). These patterns were observed across both genotypes and all development stages (Fig. 5C and Supplemental Fig. S12), suggesting the methylation levels in promoter regions were generally associated with transcriptional silencing while the methylation levels of gene body regions were more often positively associated with gene expression levels.

### Differential DNA methylation between different *P. hallii* inflorescences

**Figure 6.**
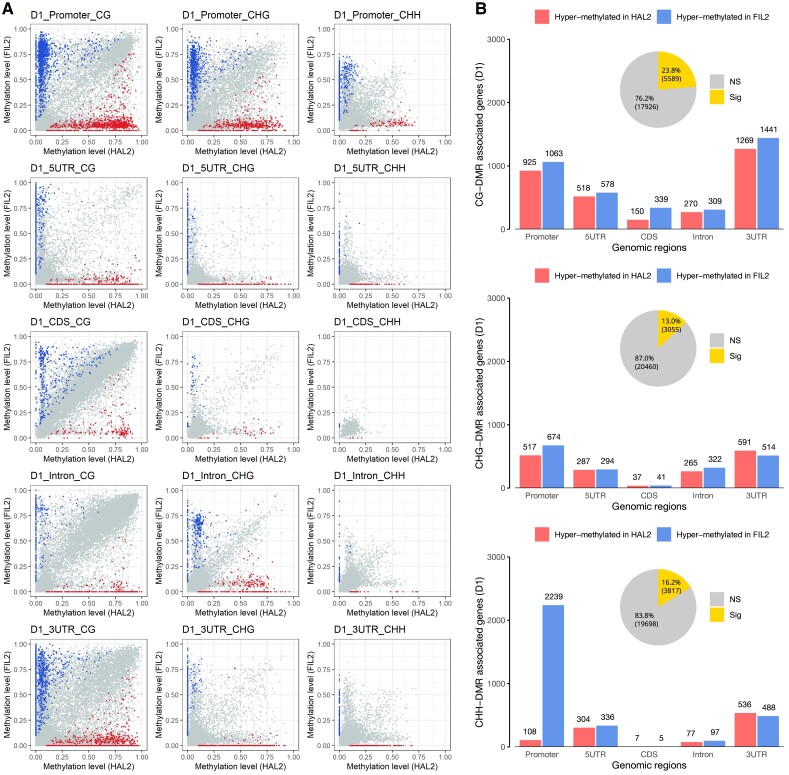
Differential DNA methylation regions between HAL2 and FIL2 inflorescences. **A)** Pairwise comparisons of methylation levels from one-to-one putative ortholog pairs between HAL2 and FIL2 D1 inflorescence in CG, CHG, and CHH contexts across 5 different genomic features. **B)** A number of differentially methylated genes between HAL2 and FIL2 D1 inflorescence in CG, CHG, and CHH contexts across 5 different genomic features are shown in bar plots. The total number of differentially methylated genes in each context is shown in the associated pie chart. In the pie charts, “NS” refers to nonsignificant methylation difference, while “Sig” refers to significant methylation difference (a cut-off of <0.01 *q*-value and >0.1 methylation change was used to identify significant methylation difference). The data for the comparison between HAL2 and FIL2 at D4 inflorescence are given in Supplemental Fig. S13. The comparison between D1 and D4 inflorescence in both HAL2 and FIL2 background is given in Supplemental Fig. S16.

To determine DMRs, we compared methylation levels across different genomic regions in one-to-one putative orthologs between 2 genotypes (HAL2 vs. FIL2) or 2 development stages (D1 vs. D4). A gene with a significantly different proportion of methylation in any of the 3 methylation contexts across at least 1 annotated feature was considered a differentially methylated gene (DMG) (*q* < 0.01, methylation level change > 0.1, see method for details). A total of 10,509 DMGs were detected between HAL2 and FIL2 across development stages: 8,414 of them were from the earliest stage, and 8,073 of them were from the late stage (Fig. 6; Supplemental Fig. S13; and Supplemental Table S6). We found 5,589 (23.8%) DMGs in the CG context, 3,055 (13.0%) DMGs in the CHG context, and 3,817 (16.2%) DMGs in the CHH context at the early stage (Fig. 6 and Supplemental Table S6). Similarly, a total of 5,035 (21.4%) DMGs in the CG context, 2,962 (12.6%) DMGs in the CHG context, and 4,066 DMGs in the CHH context were detected at the late stage (Supplemental Fig. S13 and Supplemental Table S6). In addition, we detected 4,047 differentially methylated intergenic regions (2,878 in the CG context, 2,849 in the CHG context, and 1,042 in the CHH context) at the early stage and 3,790 differentially methylated intergenic regions (2,628 in the CG context, 2,726 in the CHG context, and 847 in the CHH context) at the late stage (Supplemental Fig. S14 and Supplemental Table S7). Notably, our analysis revealed that the flanking regions of genes (e.g. promoter, 5′UTR, and 3′UTR) are more frequently differentially methylated than the regions within genes (e.g. exons and introns) (Fig. 6 and Supplemental Fig. S13). Intriguingly, we found a genome-wide bias of CHH hypermethylation in the promoter region in FIL2 (Fig. 6 and Supplemental Figs. S13 and S15), suggesting the potential role of CHH methylation in inflorescence divergence in *P. hallii*. Further, we compared the methylation levels between early and late stages of inflorescences in each genotype. We only detected 1,189 (5.1%) DMGs associated with HAL2 inflorescence development and 1,017 (4.3%) DMGs associated with FIL2 inflorescence development (Supplemental Fig. S16 and Supplemental Table S8), suggesting that methylation levels are relatively stable during inflorescence development in *P. hallii*.

### The patterns of DMR-associated DEGs evolution during inflorescence divergence

**Figure 7.**
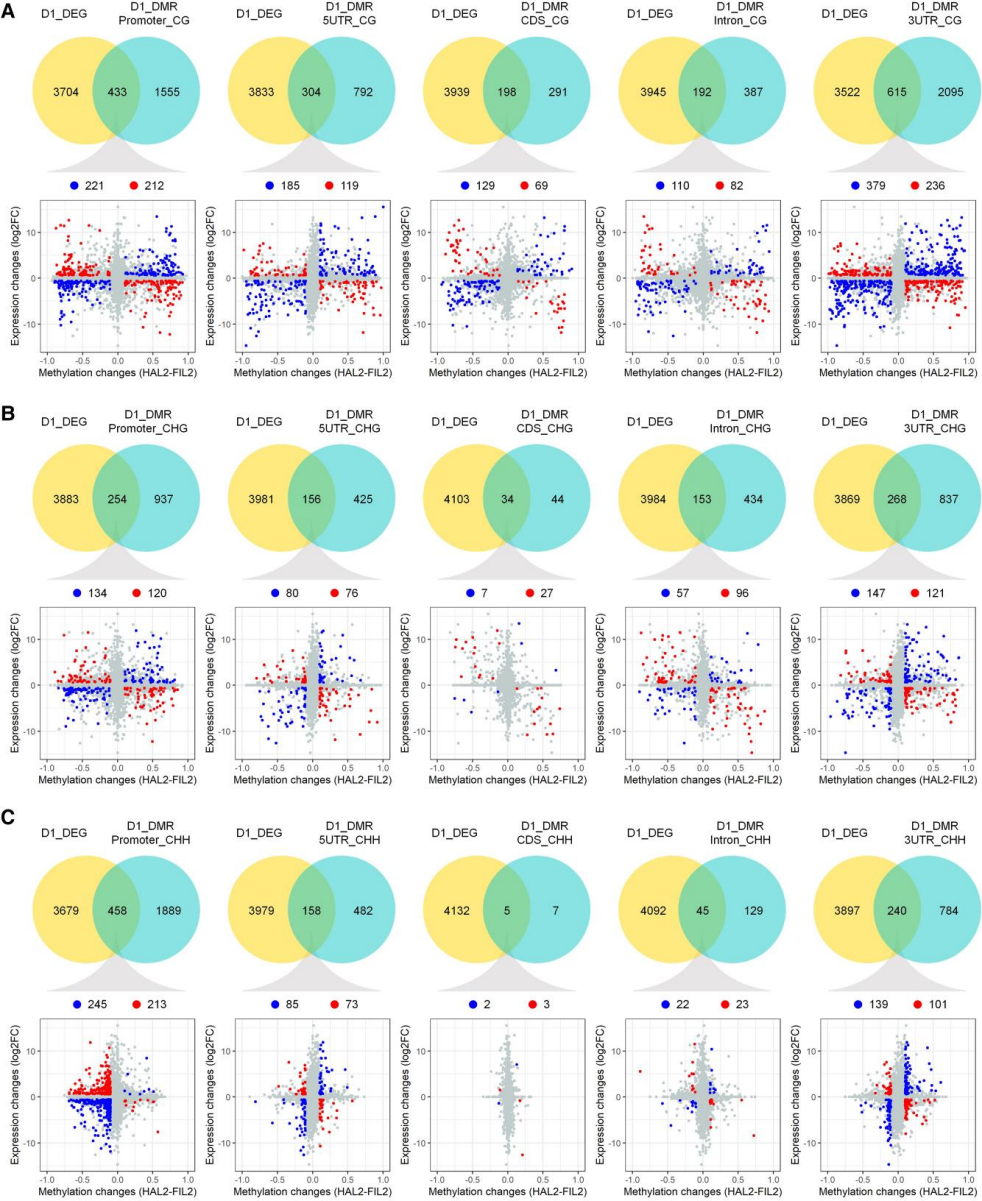
Association of differentially methylated genes with DEGs. Venn diagrams depicting the number of DEGs (left circle, DEGs) and DMR-associated genes (right circle, DMRs) between HAL2 and FIL2 D1 inflorescence in CG **(A)**, CHG **(B)**, and CHH **(C)** contexts across 5 different genomic features; 2D scatter plots depict the association of DEGs and DMRs in CG **(A)**, CHG **(B)**, and CHH **(C)** contexts across 5 different genomic features. The *x*-axis represents relative gene expression change (log_2_-fold change), while the *y*-axis represents relative methylation change (HAL2 subtracted from FIL2). The data for the association of differentially methylated genes with DEGs at D4 inflorescence are given in Supplemental Fig. S18.

To understand the role of DNA methylation in driving the expression of genes involved in inflorescence diversity, we identified DMR-associated DEGs between the ecotypes by joining the results of our methylome and transcriptome datasets. DMR-associated DEGs analysis was conducted on a set of 7,843 genes with divergent expression that were differentially expressed under stringent criteria (*q* < 0.01, fold change >1.5) in the stage-by-stage contrast analysis (Supplemental Table S2). In these criteria, a total of 1,745 and 2,180 genes at D1 and D4 stages, respectively, were identified as DMR-associated DEGs between HAL2 and FIL2 inflorescences (Supplemental Fig. S17), suggesting that more than one-third of DEGs were associated with the methylation changes (42.2% in D1 and 38.6% in D4). Among them, 1,279 genes in the CG context, 714 genes in the CHG context, and 802 genes in the CHH context were detected as DMR-associated DEGs at D1 stage (Supplemental Fig. S17). Similarly, 1,488 genes in the CG context, 827 genes in the CHG context, and 1,083 genes in the CHH context were detected as DMR-associated DEGs at D4 stage (Supplemental Fig. S17). Overall, DMR-associated DEGs with CG context differential methylation were more abundant than other sequence contexts. We observed that a larger fraction of DMR-associated DEGs had differences in methylation at the flanking regulatory regions, especially the promoter and 3′UTR regions (Fig. 7 and Supplemental Fig. S18). We noticed that CHH hypermethylation in the FIL2 promoter regions could be associated with either gene activation or repression (Fig. 7 and Supplemental Fig. S18). Intriguingly, we did not observe a simple pattern between the direction of differential methylation and differential gene expression at both stages of inflorescence development (Fig. 7 and Supplemental Fig. S18). The trend between the direction of differential methylation and differential gene expression could be positive or negative in all 3 sequence contexts located in the 5 different genic regions (Fig. 7 and Supplemental Fig. S18). Similar findings are reported in recent studies (Rajkumar et al. 2020; Li et al. 2021), suggesting the complex role DNA methylation plays in gene expression regulation.

**Figure 8.**
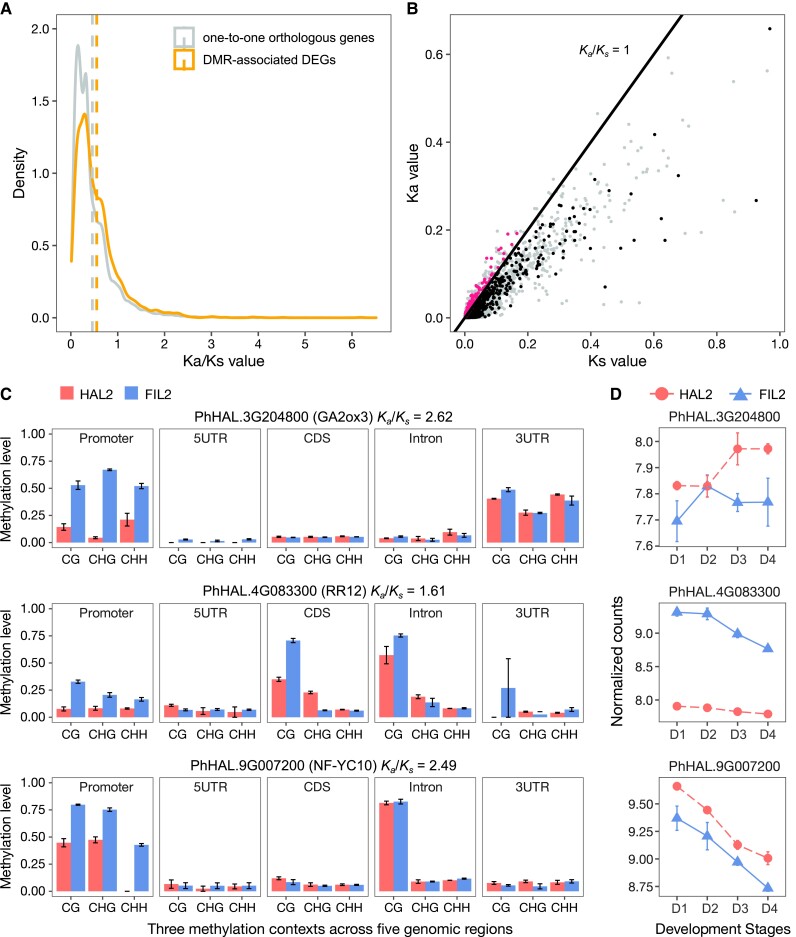
Evolution of DMR-associated DEG pairs. **A)** and **B)** The *K_a_*/*K_s_* value distribution of gene pairs from DMR-associated DEGs and one-to-one putative orthologs between HAL2 and FIL2. **A)** The mean values are indicated by the dashed line. **B)** DMR-associated DEGs with *K_a_/K_s_* ratio larger than 1. The solid line marks *K_a_*/*K_s_* = 1. **C)** Differential methylation patterns of CG, CHG, and CHH contexts across 5 different genomic features for DMR-associated DEG genes that are putatively positively selected. In all panels, the bar plots and error bars are the average values and *SE*, respectively, based on methylation level from 3 replicates. **D)** Expression patterns of DMR-associated DEG genes that are putatively positively selected. The *x*-axis represents 4 developmental stages, while the *y*-axis represents normalized counts using variance stabilizing transformation in DEseq2. In all panels, the points and error bars are the average values and *SE*, respectively, based on normalized counts of 3 RNA-seq replicates.

To explore protein evolution associated with DMR-associated DEGs, we compared the *K_a_*/*K_s_* ratio (non-synonymous substitutions per non-synonymous sites/synonymous substitutions per synonymous sites) for HAL2 and FIL2 gene pairs between DMR-associated DEGs and the genome-wide pattern for one-to-one putative orthologs. The *K_a_*/*K_s_* ratio of DMR-associated DEGs and one-to-one putative orthologs centered around a peak at 0.55 and 0.46 (Fig. 8A), respectively. No statistically significant difference in the *K_a_*/*K_s_* ratio distribution was observed between DMR-associated DEGs and the genome-wide backgrounds. We observed that only 302 (∼12.6%) of DMR-associated DEGs pairs have *K_a_*/*K_s_* ratios > 1 (Fig. 8B and Supplemental Table S9), suggesting that the majority of DMR-associated DEGs are evolving under purifying selection. Among the DMR-associated DEGs under positive selection, we identified candidates involved in hormone pathways, including the putative orthologs of *GIBBERELLIN 2-OXIDASE 3* (*GA2ox3*) and *RESPONSE REGULATOR 12* (*RR12*) (Fig. 8C). These genes have been shown to play a role in gibberellin catabolism and cytokinin signaling, respectively (Bolduc and hake 2009; Dai et al. 2017). Moreover, we detected the putative ortholog of *NUCLEAR FACTOR Y, SUBUNIT C10* (*NF-YC10*), which is associated with flowering time, inflorescence regulation, and seed size in rice (Fig. 8C) (Jia et al. 2019; Zhang et al. 2019). Among these genes, the putative ortholog of *GA2ox3* was differentially expressed with strictly additive genotype effects, while the putative orthologs of *RR12* and *NF-YC10* were differentially expressed with independent genotype and development effects (Fig. 8D). Differential methylation and expression of these positively selected genes may play an important role in the evolution of *P. hallii* inflorescences.

## Discussion

The inflorescence branching system of a plant species influences the number of flowers and seeds the plant produces. This, in turn, affects the reproductive success of plants through their life history strategies, as well as the economic potential of the crops. Genome-wide gene expression and DNA methylation analyses are now widely used to study the genetic and epigenetic mechanisms of inflorescence development from a variety of important crops (Furutani et al. 2006; Wang et al. 2010; Li et al. 2012; Eveland et al. 2014; Feng et al. 2017; Parvathaneni et al. 2020; Sun et al. 2020). Despite ever-increasing knowledge of grass inflorescence development (Kellogg 2022), an effective model of inflorescence patterning from wild species without a domestication history is still lacking. *P. hallii* is a native perennial C_4_ grass with a highly diverse and complex inflorescence with a striking divergence between ecotypes adapted to contrasting habitats (Lowry et al. 2015; Palacio-Mejia et al. 2021). To date, *P. hallii* has been developed as a complementary diploid model in parallel with domesticated crops and other C_4_ perennial grasses (Lovell et al. 2018). A systematic comparison of inflorescence transcriptome and DNA methylome for *P. hallii* ecotypes should provide insights into the molecular mechanisms leading to their divergent inflorescences.

### Divergence and heterochronic expression of inflorescence development in *P. hallii*

Previous transcriptomic studies have revealed the regulatory modules of young inflorescence development in major crops including rice, maize, and wheat (Furutani et al. 2006; Wang et al. 2010; Eveland et al. 2014; Harrop et al. 2016; Feng et al. 2017). These studies provide resources to identify potential targets for genetic engineering and overall crop improvement. However, most of these studies performed experiments within a single genetic background and, therefore, provide limited information about the evolution of gene regulatory networks associated with traits under selection. Using *P. hallii* as a model wild perennial grass, we designed a 2-factor factorial experiment to understand gene expression divergence and development. Despite only ∼1.08 Mya of divergence (Lovell et al. 2018), we demonstrated that the vast majority of genes (14,270, 73.8%) exhibited significant expression divergence between HAL2 and FIL2 inflorescence or significant expression level changes across development. Among them, we detected a considerable number of genes (5,078, 26.3%) that exhibit a significant interaction between development and genotype, suggesting the potential role of heterochronic expression during the development of inflorescence evolution in *P. hallii*. Heterochronic change is an alteration in the timing of developmental programs during evolution, which are known to contribute to the evolution of inflorescence architecture (Buendia-Monreal and Gillmor 2018). In grasses, the inflorescence branching systems are determined by the timing of phase transition from the branch meristem to the spikelet meristem (Kyozuka et al. 2014). Delays in the spikelet meristem specification result in more complex and larger inflorescences (Kyozuka et al. 2014). In our study, we observed heterochronic shifts for interaction genes potentially controlling flowering time, organ development, and hormone pathways. For example, *PRR37* is a key component in the core circadian feedback loop controlling flowering time, inflorescence architecture, and adaptation (Koo et al. 2013; Yan et al. 2013). Our findings suggested that the putative ortholog of *PRR37* had a greater expression in FIL2 than HAL2 at an earlier developmental stage (Fig. 3B). The *AP2* family gene, *SNB*, has been identified as a heterochronic gene controlling the transition from spikelet meristem to floral meristem and the floral organ development (Lee et al. 2007; Wang et al. 2015). Our data show that the putative ortholog of *SNB* had a greater expression in FIL2 than HAL2 at a later developmental stage (Fig. 3B). The *cytokinin oxidases*, *CKXs*, have been observed to regulate cytokinin accumulation in inflorescence meristems and the number of reproductive organs (Ashikari et al. 2005; Bartrina et al. 2011). Our results identified 2 putative orthologs of *CKXs* (*CKX1* and *CKX5*) with opposite predominant patterns and heterochronic changes (Fig. 3B). Moreover, we identified a significantly enriched GO term of “response to cytokinin” (GO:0009735) in Cluster 1 of interaction genes. We observed that the majority of these cytokinin response genes have potential heterochronic expression changes, that is, a greater expression in HAL2 than FIL2 at a later developmental stage. The heterochronic expression of genes involving in cytokinin signaling and catabolism pathways may play a role in the divergence of the inflorescence development between the *P. hallii* ecotypes. It was noticed that a large number of genes (7,285) have been identified to have independent genotype and development effects. We found a significantly enriched GO term of “response to auxin” (GO:0009733) in Cluster 1 of this category gene. It is well established that auxin signaling plays a role in the formation of axillary meristems and inflorescence development (Deveshwar et al. 2020). Further, we observed several putative orthologs of *MADS* transcription factors in an enriched GO term of “flower development” (GO:0009908) in Cluster 4 of this category gene. These *MADS* genes expressed higher in FIL2 relative to HAL2, with a general pattern of decreasing expression with development. It was known that *MADS* genes control inflorescence branching systems via the regulation of meristem identification and development in grasses (Liu et al. 2013; Kyozuka et al. 2014). Despite not being identified as interaction effects, these genes were still thought to be playing a role in the expression divergence between HAL2 and FIL2 inflorescence.

### DNA methylation and the evolution of inflorescence development in *P. hallii*

In this study, we performed whole-genome bisulfite sequencing to understand the role of DNA methylation during inflorescence development and architecture divergence. In our methylome study in *P. hallii*, we found that the overall methylation levels from tissues across different genotypes and development stages were similar in each context. The proportions of methylated cytosines in CG, CHG, and CHH contexts were 66%, 49%, and 3.1%, respectively. For comparison, values reported in *Arabidopsis* are 30.5% for CG, 10.0% for CHG, and 3.9% for CHH, in rice are 58.4% for CG, 31.0% for CHG, and 5.1% for CHH, and in maize are 86% for CG, 74% for CHG, and 5.4% for CHH (Niederhuth et al. 2016). These results suggested that *P. hallii* has an intermediate level of DNA methylation. Coincident with previous findings, we observed a strong positive association between CG and CHG methylation with gene density, suggesting the role of DNA methylation in the establishment and maintenance of centromeric and pericentromeric heterochromatin regions. We also found a positive association between CHH methylation and gene density (except in HAL2-D4 samples). Previous studies have shown that methylation at CHH sites is kept high in rice reproductive organs compared with vegetative tissues (Higo et al. 2020). Our analysis of the relationship between gene expression and DNA methylation level suggested that non-expressed and lowly expressed genes showed higher CG and CHG methylation levels in their proximal regulatory regions, while genes expressed at high levels were highly CG methylated within their gene body regions. These patterns are similar to recent results reported in chickpea and pineapple (Rajkumar et al. 2020; Shi et al. 2021), suggesting the conserved antagonistic role of CG methylation in gene expression regulation in the regulatory and gene body regions.

Previous studies have shown that mapping bias to a single genome can introduce clear and substantial quantification bias in the identification of DMRs (Wulfridge et al. 2019). Notably, most methylome studies align to a single reference genome to identify DMRs between different genotypes due to the limitation of genomic resources (Li et al. 2012; Rajkumar et al. 2020). Our previous studies have developed reference genomes for HAL2 and FIL2 and investigated the genome size divergence between the 2 ecotypes (487 Mb in HAL vs. 535 Mb in FIL2) (Lovell et al. 2018). Here, we observed a dramatic drop in mapping efficiencies from alignments to individual genomes (HAL2 to HAL2 and FIL2 to FIL2, ∼75%) compared with alignments to divergent genomes (HAL2 to FIL2 and FIL2 to HAL2, ∼30%). After mapping reads to their own individual genome references, we found that almost half of the one-to-one putative orthologs (10,509) are differentially methylated in at least 1 feature of genomic regions between HAL2 and FIL2 across inflorescence development. This degree of widespread natural variation in DNA methylation was also observed in a diverse panel of Arabidopsis and maize (Kawakatsu et al. 2016; Xu et al. 2020). Interestingly, previous studies showed that differential methylation primarily occurs within gene body regions (Rajkumar et al. 2020). However, we observed that flanking regulatory regions, including promoter, 5′UTR, and 3′UTR, are more frequently differentially methylated than the regions within gene body regions. One explanation for this conflicting pattern could be the difference in alignment strategy, as most of these studies mapped reads from different genotypes to 1 genome reference and probably induced quantification bias in the highly variable regulatory regions. Intriguingly, we observed a significant bias of CHH hypermethylation in the promoters of FIL2 genes. Unlike CG and CHG methylation, CHH methylation is more dynamic and is deposited de novo every generation (Martin et al. 2021). This genome-wide pattern of CHH hypermethylation in the promoter regions of FIL2 genes might be associated with population expansion in *P. hallii* from the coast to inland and may contribute to local adaptation through gene expression regulation related to morphological and physiological change. Although tissue or developmental stage-specific methylation patterns have been mentioned in some studies (Huang et al. 2019), we only detected a few DMGs between the 2 stages of development. Considering that a large number of one-to-one putative orthologs are differentially expressed across development stages, other processes beyond differential methylation are likely to be involved in the observed expression variation.

Finally, we detected 2,911 DMR-associated DEGs between HAL2 and FIL2 across inflorescence development. The relationship between the direction of differential methylation in different sequence contexts and differential gene expression is not simple, including both positively and negatively associated patterns. This complex pattern was also observed in recent studies in other species (Rajkumar et al. 2020; Li et al. 2021). Although recent evidence from population-level studies suggested that selection on DNA methylation could be weak, differential methylation of key development genes is associated with phenotypic variation (Xu et al. 2020). In our study, we identified the putative orthologs of *GA2ox3* and *RR12* as DMR-associated DEGs. The gibberellin catabolism gene is a direct target of *KNOTTED1* (*KN1*), a key transcription factor involved in the establishment and maintenance of plant meristems (Bolduc and hake 2009). It was reported that *RR12* functions as a molecular link between cytokinin signaling and the expression of shoot meristem genes *WUSCHEL* (*WUS*) (Dai et al. 2017). Interestingly, both of the hormone genes are potentially under positive selection. Functional validation of DMR-associated DEGs in future studies will provide insights into the evolutionary processes driving the divergence of inflorescence morphology in *P. hallii*.

## Materials and methods

### Plant materials and sample collection

Hall's panicgrass (*P. hallii*) genotypes, HAL2 (*P. hallii var. hallii*, upland ecotype) and FIL2 (*P. hallii var. filipes*, lowland ecotype), were grown in a growth chamber at the University of Texas at Austin with 26 °C day/22 °C night temperature and 12-h photoperiod. Plants were grown in 3.5 in. square pots with a 6:1:1 mixture of Promix:Turface:Profile soil. The first fully emerged inflorescence was photographed and used to measure the primary branch number, secondary branch number, and spikelet number as previously described (Wang et al. 2015). The seeds were harvested after maturity and dried at a temperature of 37 °C until the seed weight was stable. The dried seeds were photographed and weighed for the 100-seed-weight (mg) value. Phenotypic values are averages from 8 replicates showing uniform growth. Young panicle tissues were collected under a dissection microscope and the developmental stages were determined according to the lengths (0.1 to 0.2 cm for D1 stage, 0.5 to 1 cm for D2 stage, 4.5 to 5.5 cm for D3 stage, and 9 to 11 cm for D4 stage). Tissues for D1 and D2 stages were taken from at least 50 plants and pooled for each biological replicate. Tissues for D3 and D4 stages were taken from at least 15 plants and pooled for each biological replicate. All samples were harvested from 17:00 to 18:00 of the day and immediately flash frozen in liquid nitrogen and stored at −80 °C. Three biological replicates from D1 to D4 stages were used for RNA extraction and transcriptome study. Three biological replicates at D1 and D4 stages were used for DNA extraction and methylome study.

### Scanning electron microscope

The inflorescences at the D1 and D2 developmental stages were dissected from plants that were collected from the greenhouse at the University of Texas at Austin. These inflorescences were then fixed in a PFA + GA buffer (phosphate-buffered 4% paraformaldehyde + 4% glutaraldehyde, v/v for all solutions) overnight. After removing the unbound fixative, specimens were immersed in 1% OsO4 (osmium tetroxide) overnight followed by the OTOTO method as implemented before (Bess et al. 2005). The specimens were then dehydrated through graded alcohols (50, 70, 90, 95, 100, 100% ethanol, 1:1 HDMS:ethanol [hexamethyldisilazane:ethanol], 100% HDMS). The air-dried samples were mounted on stubs with adhesive tape and sputter coat and were then imaged in a Zeiss Supra40 SEM at 10 kV in the Microscopy and Imaging Facility at the University of Texas at Austin.

### Sequence analysis

Totally, 19,332 one-to-one putative orthologs were identified in a previously published *P. hallii* genomic study (Lovell et al. 2018). The synonymous substitution rates (*K_s_*), non-synonymous rates (*K_a_*), and non-synonymous to synonymous substitution ratios (*K_a_*/*K_s_*) of all one-to-one putative ortholog pairs of HAL2 and FIL2 were estimated by using the “simple Ka/Ks calculator” function from TBtools (Chen et al. 2020).

### RNA extraction and RNA-seq library preparation

For RNA preparation, inflorescence samples from 4 development stages were homogenized to a fine powder using a prechilled mortar and pestle under liquid nitrogen. Total RNA was isolated using the TRIzol kit (Invitrogen) and samples were treated with DNase I (Invitrogen) to remove contaminating genomic DNA. RNA-seq libraries were prepared and sequenced at the Department of Energy Joint Genome Institute (Lawrence Berkeley National Laboratory, Berkeley). Briefly, the integrity and concentration of the RNA preparations were checked initially using Nano-Drop (Nano-Drop Technologies) and then by BioAnalyzer (Agilent Technologies). Total RNA-seq libraries were prepared using Illumina's TruSeq Stranded mRNA HT sample prep kit utilizing poly-A selection of mRNA. Sequencing was performed on the Illumina HiSeq 2500 platform using HiSeq TruSeq SBS sequencing kit, following a 2 × 150 indexed run recipe.

### RNA-seq data analyses

Paired-end RNA-seq 150-bp reads were quality trimmed (*Q* ≥ 25) and reads shorter than 50 bp after trimming were discarded. High-quality filtered reads were aligned to their own reference genomes, *P. hallii* HAL v2.1 and *P. hallii* v3.1 (https://phytozome-next.jgi.doe.gov/), using GSNAP with a maximum of 4 mismatches. The HTseq-count was used to generate raw gene counts, and only reads that were uniquely mapped to 1 annotated gene were counted. To filter the genes with low expression and compare the diverged transcriptome assemblies, only one-to-one putative orthologs with counts-per-million above 0.5 (correspond to a count between 8 and 10 for different library sizes) in at least 3 samples were retained for further analysis. Principal component analysis and specific gene expression patterns were performed with *vst* normalized expression counts and visualized using the ggplot2 package. DEGs with main effects and developmental-specific effects were determined as previously described (Weng et al. 2019). Briefly, to study the additive and interaction effects of genotype and development stage, we determined differential gene expression using statistical testing via likelihood ratio tests in DEseq2 (Love et al. 2014). We used a factorial linear model to test the following: (a) genotype additive effect by comparing the difference in deviance between the 2-factor additive model (Genotype + Development stage) and a reduced model (Development stage) formula; (b) the effect across development stages by comparing the difference in deviance between the 2-factor additive model (Genotype + Development stage) and a reduced model (Genotype); and (c) interaction effect by comparing the difference in deviance between the full model (Genotype + Development stage + Genotype × Development stage) and an additive reduced model (Genotype + Development stage). Multiple testing was controlled by the *q*-value transformation of the likelihood ratio test *P*-value and genes with expression divergence were determined by significant genotype and/or interaction effects (*q* < 0.01). To further study developmental stage-specific effects, we conducted a linear model fit to a set of 4 HAL2-FIL2 contrasts with genes exhibited genotype and/or interaction effects, 1 at each development stage, through a custom contrast analysis pipeline in DEseq2 with the calculation of log_2_-fold change values and adjusted *P*-value (Weng et al. 2019). The *vst* transformed counts of genes were used to plot the divergence expression profiles of putative ortholog pairs between HAL2 and FIL2. The profile of genotype predominant genes with developmental stage-specific information was plotted with the UpSetR package (Conway et al. 2017). The minimum centroid distance was determined with the Mfuzz package (Kumar and Futschik 2007). The *k*-mean clustering strategy was applied for each category of DEGs and the outputs were visualized using the Complex Heatmap package (Gu et al. 2016). The “GO enrichment” function of TBtools was used to perform the GO enrichment analysis of DEGs in each cluster (Chen et al. 2020).

### DNA extraction and bisulfite sequencing library preparation

A CTAB-based protocol was used for DNA extraction from D1 and D4 inflorescence samples of both genotypes (HAL2 and FIL2). The quality of DNA was determined by running on a 1.0% agarose gel electrophoresis and quantified via Nano-Drop (Nano-Drop Technologies). DNA methylome libraries were prepared from 1 *μ*g of genomic DNA and underwent bisulfite treatment using NextFlex Bisulfite-Seq Kit. The resulting bisulfite-converted DNA was PCR-amplified and ligated to adapters, with barcodes. Amplified fragments were purified using the 1.8 × AMPure XP bead to remove the small fragments. The libraries were checked for size and concentration using the Agilent Bioanalyzer instrument, followed by sequencing on the Illumina HiSeq 2500 platform at HudsonAlpha Institute.

### Bisulfite sequencing data analyses

Paired-end bisulfite sequencing 150-bp reads were trimmed using Trim Galore with default options to remove low-quality reads and adaptor sequences. To avoid mapping bias induced by divergent reference genomes, high-quality filtered reads were aligned to their own respective reference genomes, *P. hallii* HAL v2.1 and *P. hallii* v3.1 (https://phytozome-next.jgi.doe.gov/), using Bismark with options –bowtie2 –bam (Krueger and Andrews 2011). Reference genomes index files for HAL2 and FIL2 were generated from their corresponding FASTA files using the bismark_genome_preparation function. After removing duplications with the deduplicate_bismark function, BAM output files were sorted in preparation for methylation extraction using Samtools. Genome-wide cytosine reports were obtained using the bismark_methylation_extractor with options -p -ignore 5 -ignore r2 5 -ignore 3prime 2 -ignore 3prime r2 -no_overlap –comprehensive -CX. This report was used to generate the read coverage, global methylation level, and distribution of methylation level using ViewBS (Huang et al. 2018). Reads mapped to unmethylated chloroplast genome were used to calculate the frequency of cytosine conversion. For DMR analysis, only cytosines that were covered by at least 5 reads were kept for downstream analysis. We studied differential methylation between ecotypes or developmental stages using a genomic feature approach. We defined genomic regions to include promoter regions from 500-bp upstream of the transcription start site, 5′-untranslated regions (5′UTR), protein-coding regions (CDS), intron, 3′-untranslated regions (3′UTR), and intergenic regions based on the annotation of gene structure from the existing *P. hallii* genome GFF files for each respective genome. The methylation level in each genomic region was measured as the average of the proportion of all methylated cytosines in that region. The methylation levels of different genomic regions from one-to-one putative orthologs were extracted and used for DMR analysis. For each DMR contrast, we performed a Student *t*-test and calculated the *q*-value using qvalue package to control for the large number of statistical tests. We calculated the methylation changes by subtracting average methylation proportions from HAL2 to FIL2. A cut-off of <0.01 *q*-value and >0.1 methylation change was used to identify significant DMRs across 5 genomic regions and 3 methylation contexts. In a small number of cases, methylated cytosines were detected for 1 level of a contrast but not for the other (e.g. methylation was observed in only 1 ecotype or developmental state for a feature). For these genes/regions, we simply used a cut-off > 0.1 methylation proportion change to identify significant DMRs.

### Accession numbers

The RNA sequencing data are available at JGI Plant Gene Atlas (https://plantgeneatlas.jgi.doe.gov) (Sreedasyam et al. 2022). The bisulfite sequencing is available in the Sequence Read Archive (SRA) database (https://www.ncbi.nlm.nih.gov/sra/) of NCBI (BioProject ID PRJNA895698). The accession numbers of the major genes mentioned in this paper are provided in Supplemental Table S10.

## Supplementary Material

kiad209_Supplementary_Data

## Data Availability

Raw reads for the transcriptome experiment are available in the Sequence Read Archive database (https://www.ncbi.nlm.nih.gov/sra) with accession numbers from SRR19546930 to SRR19546953 (24 samples). and from SRR22265532 to SRR22265543 (methylome). Raw reads for the methylome experiment are available in the Sequence Read Archive database (https://www.ncbi.nlm.nih.gov/sra) with accession numbers from SRR22265532 to SRR22265543 (12 samples).

## Supplemental data

The following materials are available in the online version of this article.

**Supplemental Figure S1**. Scanning electron microscopy (SEM) analysis of *P. hallii* inflorescence at D1 and D2 development stages.

**Supplemental Figure S2**. Determination of the number of cluster cores for genes with interaction effects, independent genotype and development effects, strictly additive genotype effects, and strictly additive development effects.

**Supplemental Figure S3**. Stage-specific contrasts of genes with independent genotype and development effects.

**Supplemental Figure S4**. Divergence patterns of genes with independent genotype and development effects.

**Supplemental Figure S5**. Divergence patterns of genes with strictly additive genotype effects.

**Supplemental Figure S6**. Expression patterns of genes with strictly additive development effects.

**Supplemental Figure S7**. Mapping efficiencies by performing alignments of all samples to their private reference genomes.

**Supplemental Figure S8**. Global read coverage distribution of cytosine in each context for all samples.

**Supplemental Figure S9**. Global distribution of methylation levels in each context for all samples.

**Supplemental Figure S10**. Global DNA methylation profiling of HAL2 and FIL2 at D4 stage of inflorescence development.

**Supplemental Figure S11**. The expression levels of 6 gene groups for comparison to methylation level across gene regions.

**Supplemental Figure S12**. Influence of DNA methylation on gene expression of HAL2 and FIL2 at D4 stage of inflorescence development.

**Supplemental Figure S13**. Differential DNA methylation regions between HAL2 and FIL2 inflorescences at D4 stage of inflorescence development.

**Supplemental Figure S14**. Pairwise comparisons of methylation levels from paired intergenic regions between HAL2 and FIL2 inflorescence in CG, CHG, and CHH contexts at D1 and D4 stages.

**Supplemental Figure S15**. Examples with considerable CHH hypermethylation (highlighted in gray boxes) in the promoter regions in FIL2 genes.

**Supplemental Figure S16**. Pairwise comparisons of methylation levels from one-to-one putative ortholog pairs between D1 and D4 in HAL2 and FIL2 inflorescence in CG, CHG, and CHH contexts across 5 different genomic features.

**Supplemental Figure S17**. Differentially methylated and expressed genes between HAL2 and FIL2 inflorescence.

**Supplemental Figure S18**. Association of differentially methylated genes with differentially expressed genes at D4 stage.

**Supplemental Table S1**. Factorial linear modeling analyses of genotype, development, and their interaction for all expressed genes.

**Supplemental Table S2**. Significantly differentially expressed genes between HAL2 and FIL2 across 4 development stages.

**Supplemental Table S3**. Clustering analysis for genes with interaction effects, strictly additive genotype effects, strictly additive development effects, and independent genotype and development effects.

**Supplemental Table S4**. GO enrichment analysis for different clusters of genes with interaction effects, strictly additive genotype effects, strictly additive development effects, and independent genotype and development effects.

**Supplemental Table S5**. The quality and coverage of all bisulfite sequencing data.

**Supplemental Table S6**. Significantly CG-, CHG-, and CHH-differentially methylated genes between HAL2 and FIL2 at D1 and D4 stages.

**Supplemental Table S7**. Significantly CG-, CHG-, and CHH-differentially methylated intergenic regions between HAL2 and FIL2 at D1 and D4 stages.

**Supplemental Table S8**. Significantly CG-, CHG-, and CHH-differentially methylated genes between D1 and D4 stages in HAL2 and FIL2 inflorescences.

**Supplemental Table S9**. 302 DMR-associated DEGs pairs that have *K_a_*/*K_s_* ratios greater than 1.

**Supplemental Table S10**. The accession numbers of the major genes mentioned in this paper and their putative orthologs in Arabidopsis and rice.
