# Supplementary material for: Transcriptome and DNA methylome divergence of inflorescence development between 2 ecotypes in Panicum hallii
Source: Plant Physiol. 2023 Apr 5;192(3):2374–93. doi: 10.1093/plphys/kiad209 (PMC10315280; doi:10.1093/plphys/kiad209)
Supplement: kiad209_Supplementary_Data [file kiad209_supplementary_data.zip › PP2022RA01563DR1_Supplemental_Figure_R2.pdf]

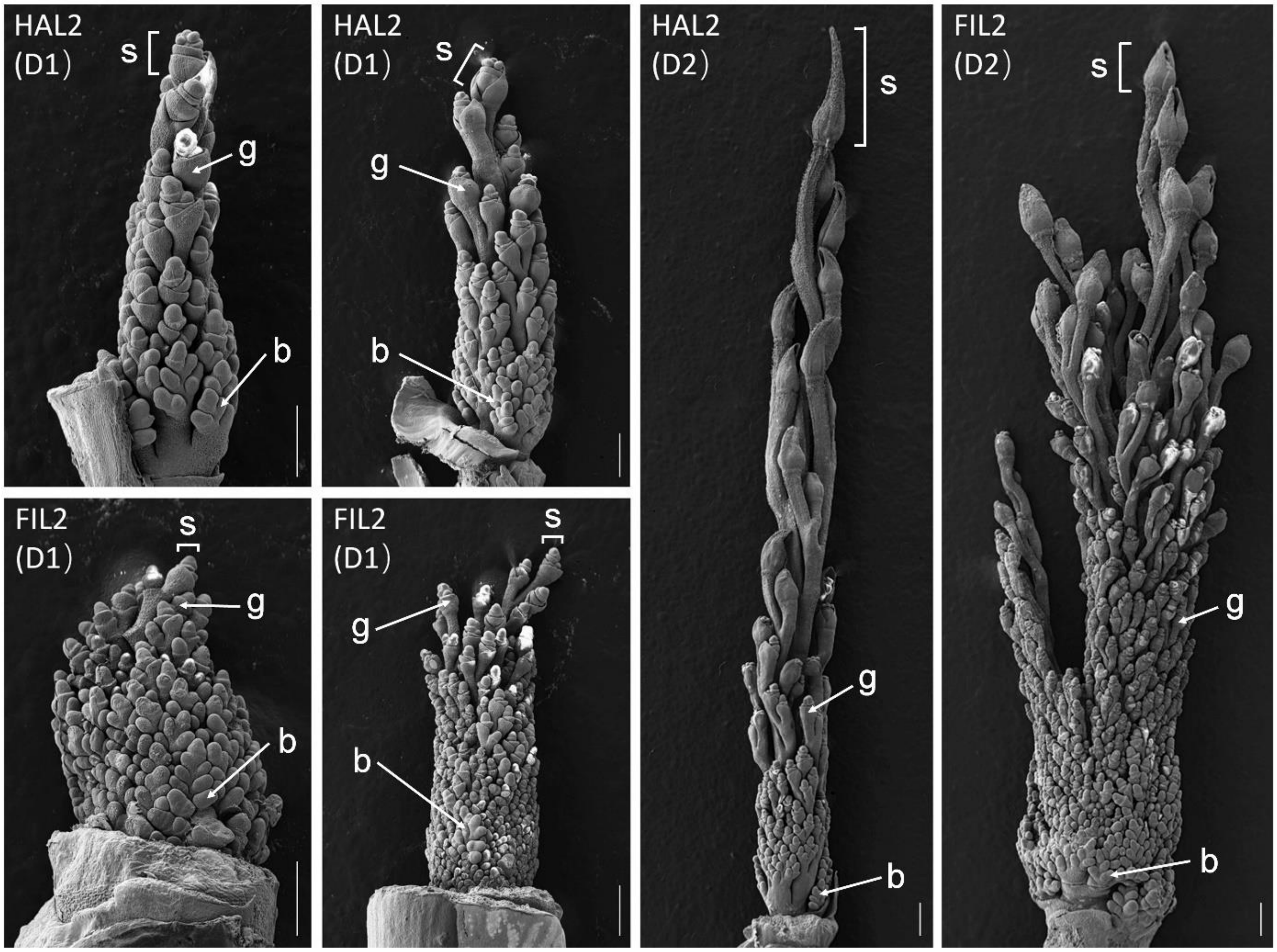

Supplemental Figure S1. Scanning electron microscopy (SEM) analysis of *P. hallii* inflorescence at D1 and D2 development stages. D1 is the inflorescence meristem stage with initiation of spikelet and glume development at the apex and branching development at the base. D2 is the late inflorescence development sage with spikelet maturing from the apex of the panicle downward. s: spikelet meristem; g: glume; b: branch meristem. Scale bars, 200 μm in all panels.

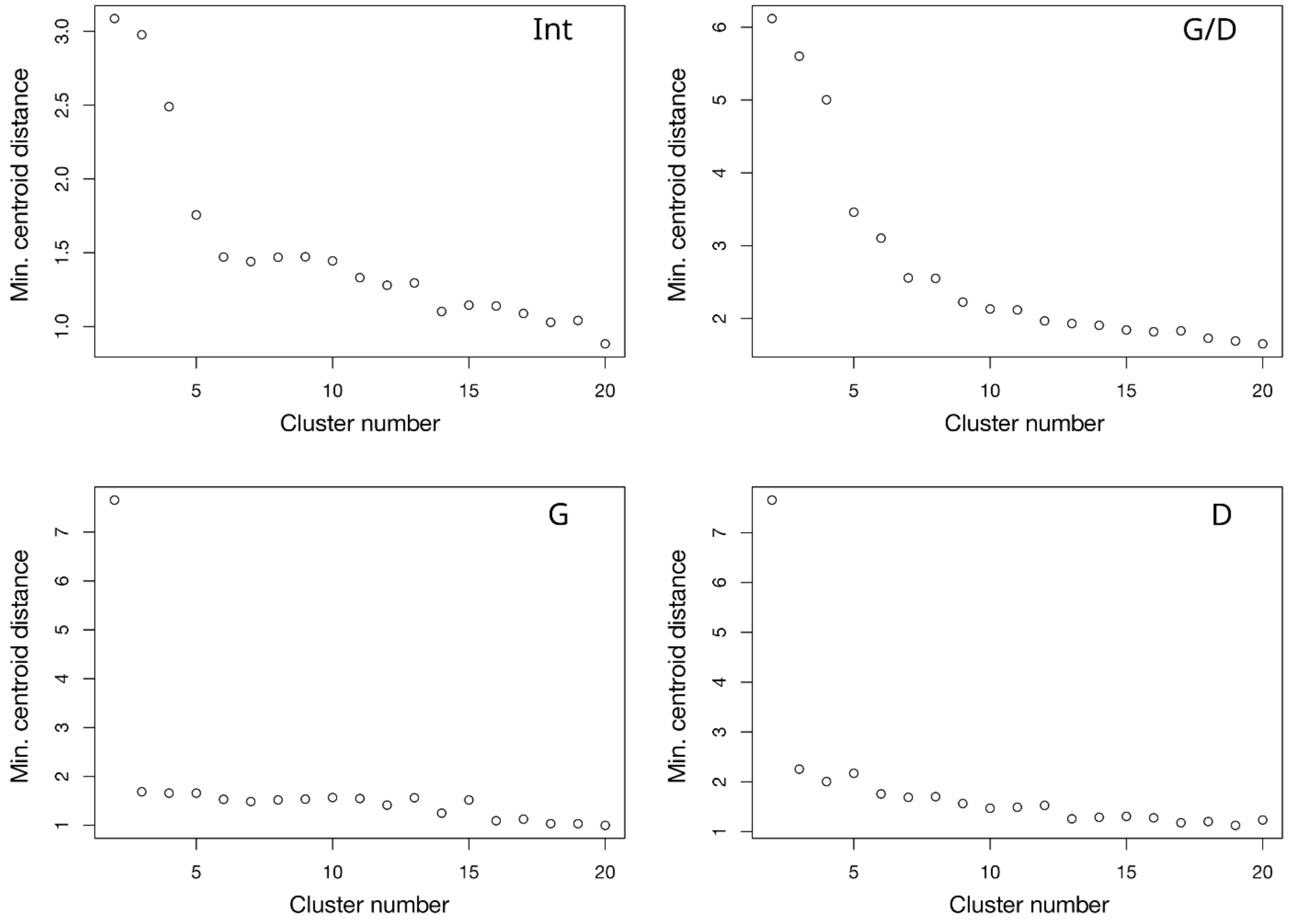

Supplemental Figure S2. Determination of the number of cluster cores for 5,078 interaction genes (Int), 7,285 genes with both genotype and development effects independently (G/D), 1,907 genes with strictly additive genotype effects (G), and 3,966 genes exhibiting strictly additive developmental effects (D). *x*-axis represents the cluster number in the test, while the *y*-axis represents the minimum centroid distance.

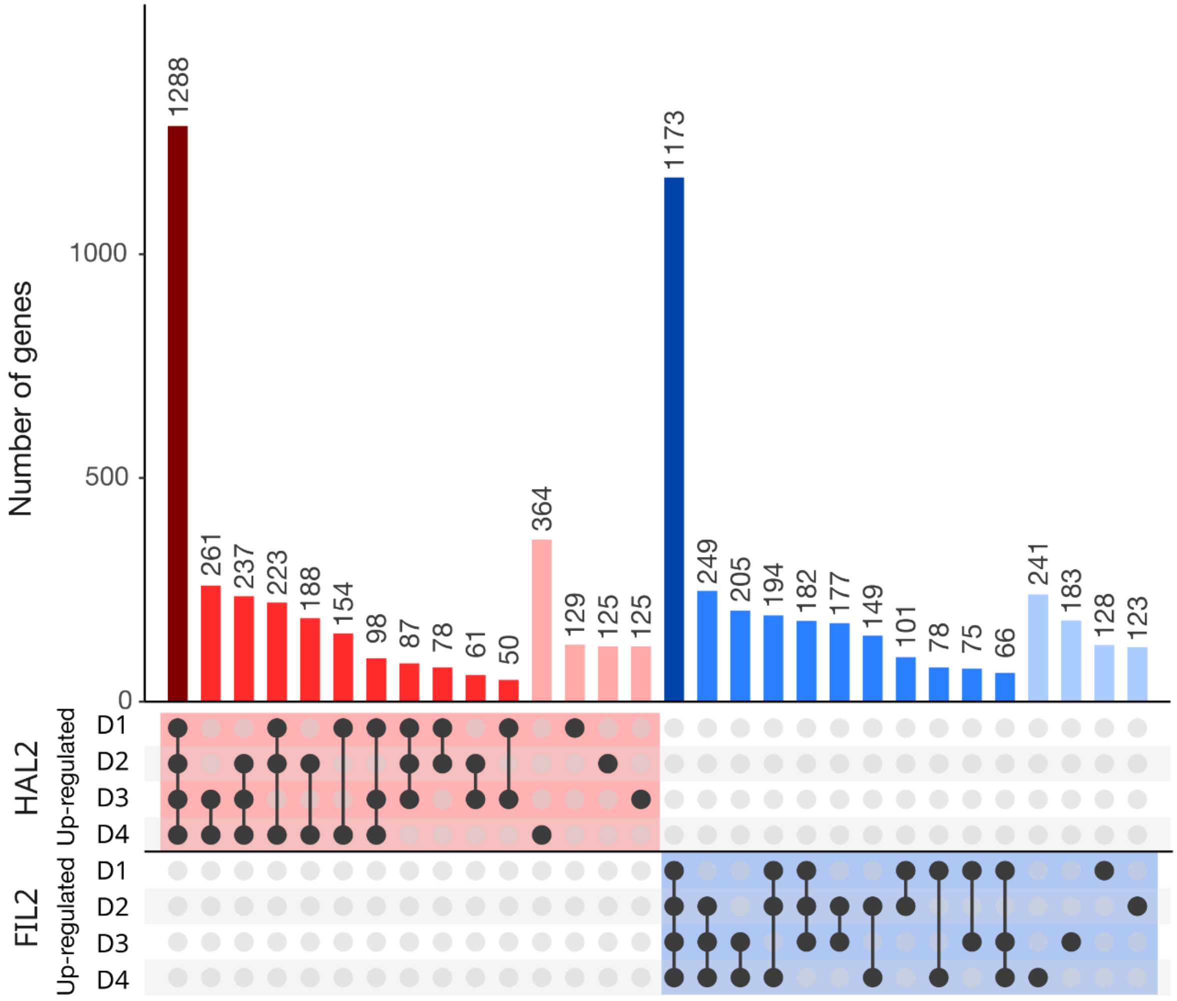

Supplemental Figure S3. Stage-specific contrasts of genes with independent genotype and development effects. Quantification of stage-specific expression of 7,285 genes with both genotype and development effects independently as HAL2 predominant (red bar on the top) and FIL2 predominant (blue bar on the top) patterns. The numbers of genes showing developmental-specific expression patterns in one or more of sampling stages are shown in black vertical bars of the figure. Black dots at the bottom of each vertical bar indicate the developmental-specific expression identified at each sampling stage. The lined dots indicate two or more sampling stages showing differential expression between two genotypes.

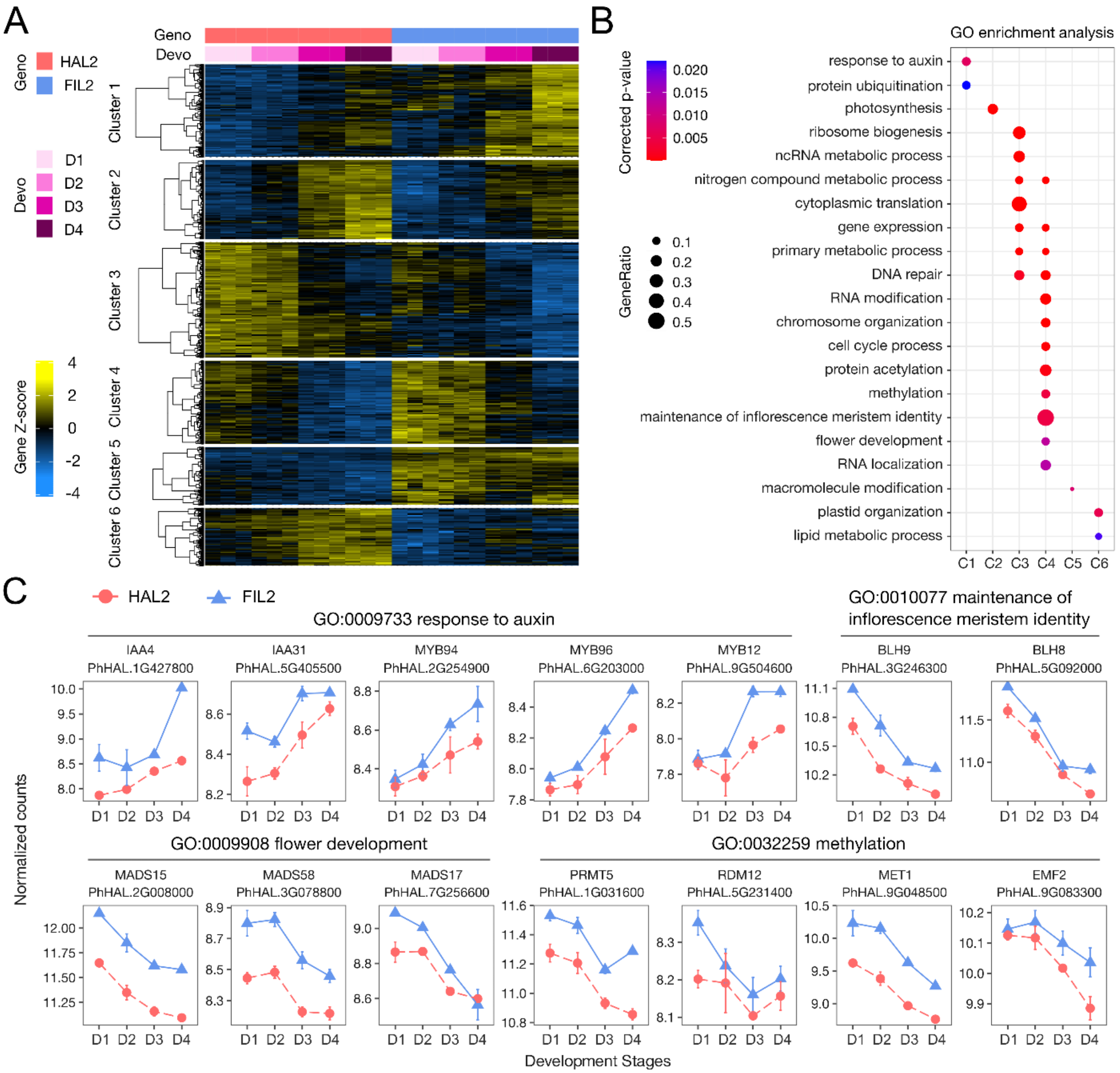

Supplemental Figure S4. Divergence patterns of genes with independent genotype and development effects. (A) Heatmaps of genes with both genotype and development effects independently between HAL2 and FIL2 across four developmental stages. Only gene expression data from 7,285 genes with both genotype and development effects independently are used for clustering. The minimum centroid distance was used to determine the number of cluster cores. The genotype and development information is added on top as color bars. (B) The dot plot of the most significantly enriched Gene Ontology (GO) terms from each cluster (*y*-axis) in 7,285 genes with both genotype and development effects independently. The size of the dots represents the number of genes in the significant differentially

expressed gene list associated with the GO term and the color of the dots represents the False Discovery Rate (FDR) corrected *p*-values (Benjamini-Hochberg method). (C) Expression of genes from enriched GO terms of “response to auxin” (GO:0009733), "maintenance of inflorescence meristem identity" (GO:0010077), "flower development" (GO:0009908), and "methylation" (GO:0032259). The *x*-axis represents four developmental stages, while the *y*-axis represents normalized counts using variance stabilizing transformation in DEseq2. In all panels, the points and error bars are the average values and *SE*, respectively, based on normalized counts of three RNA-seq replicates. The gene ID and the names of their putative orthologs are shown on the top of the expression pattern plots.

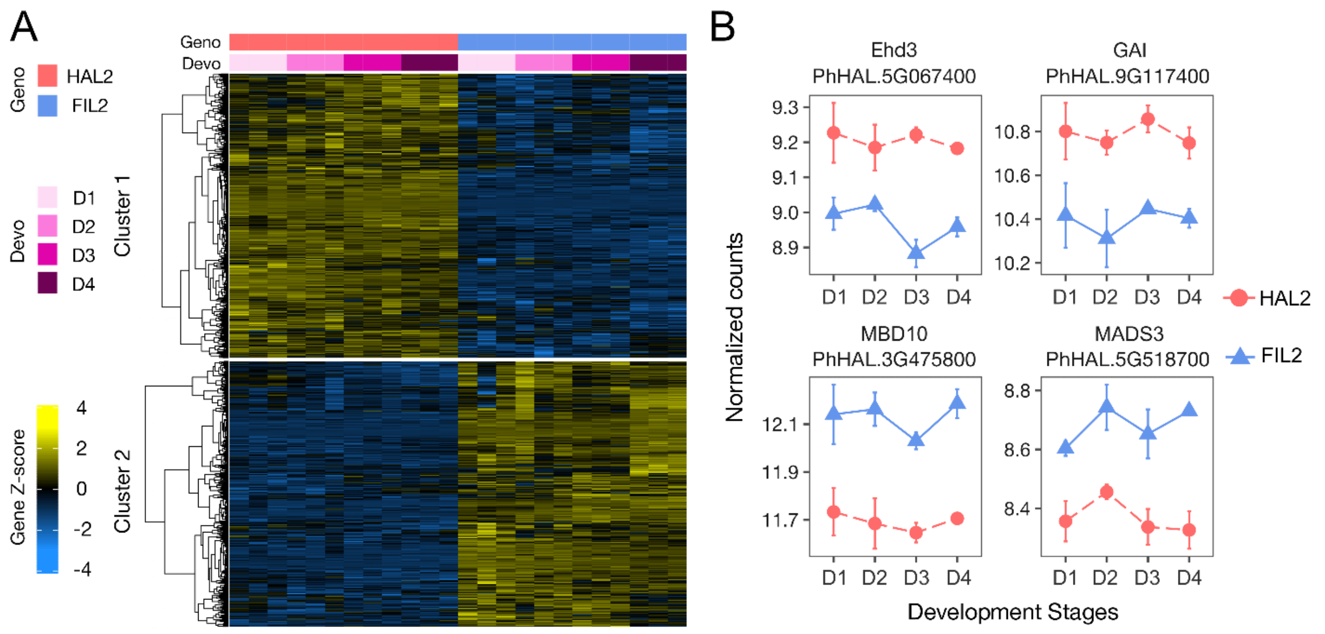

Supplemental Figure S5. Divergence patterns of genes with strictly additive genotype effects. (A) Heatmaps of gene expression with strictly additive genotype effects genes between HAL2 and FIL2 across four developmental stages. Only gene expression data from 1,907 strictly additive genotype effects genes are used for clustering. The minimum centroid distance was used to determine the number of cluster cores. The genotype and development information is added on top as color bars. (B) Expression of strictly additive genotype effects genes with HAL2 and FIL2 predominant patterns. The *x*-axis represents four developmental stages, while the *y*-axis represents normalized counts using variance stabilizing transformation in DEseq2. In all panels, the points and error bars are the average values and *SE*, respectively, based on normalized counts of three RNA-seq replicates. The gene ID and the names of their putative orthologs are shown on the top of the expression pattern plots.

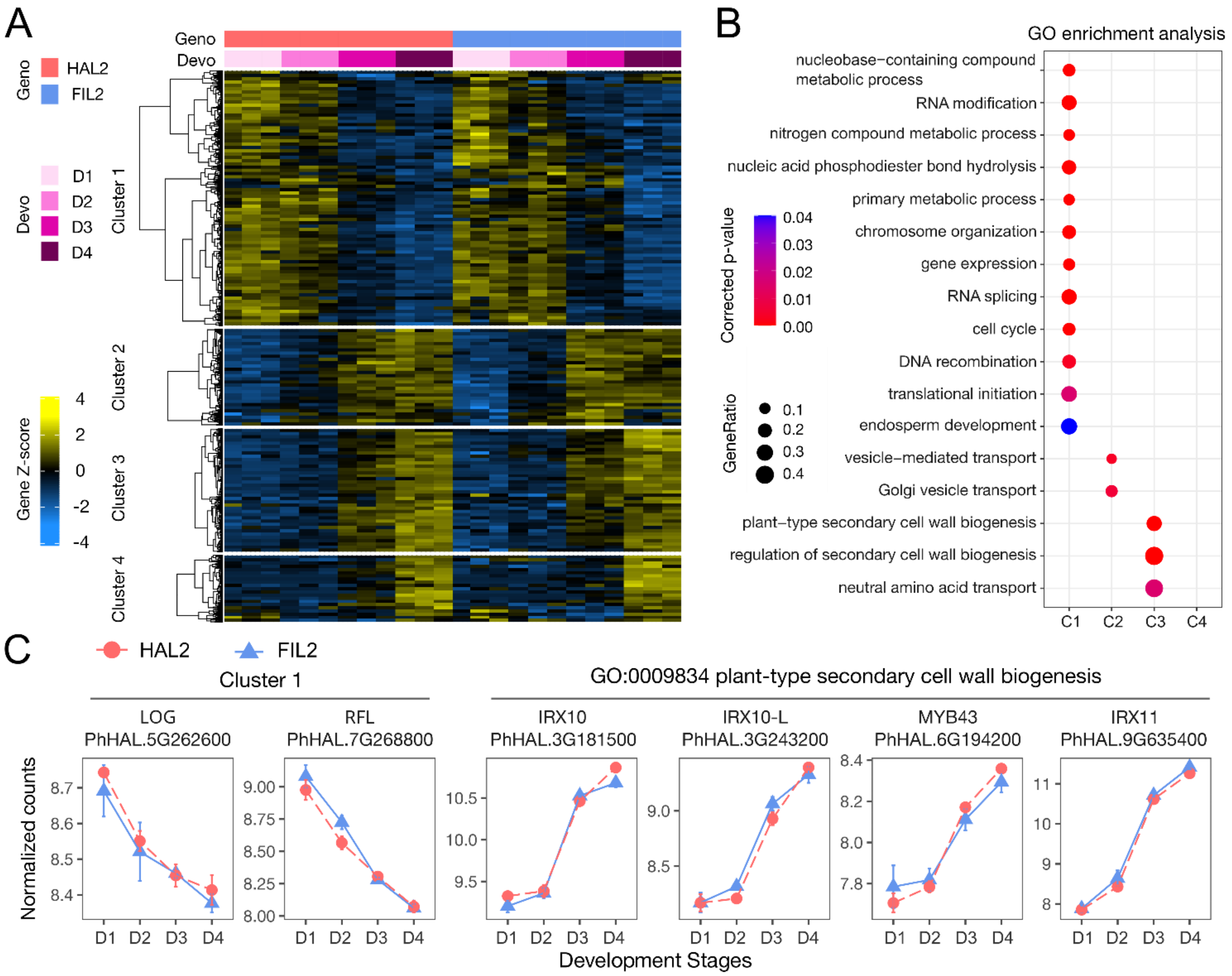

Supplemental Figure S6. Expression patterns of genes with strictly additive development effects. (A) Heatmaps of gene expression with strictly additive development effects genes between HAL2 and FIL2 across four developmental stages. Only gene expression data from 3,966 strictly additive genotype effects genes are used for clustering. The minimum centroid distance was used to determine the number of cluster cores. The genotype and development information is added on top as color bars. (B) The dot plot of the most significantly enriched Gene Ontology (GO) terms from each cluster (*y*-axis) in 3,966 strictly additive genotype effects genes. The size of the dots represents the number of genes in the significant differentially expressed gene list associated with the GO term and the color of the dots represents the False Discovery Rate (FDR) corrected *p*-values (Benjamini-Hochberg method). (C) Expression of strictly additive development effects genes from the cluster 1 and genes from the enriched GO term of "plant-type secondary cell wall biogenesis" (GO:0009834). The *x*-axis represents four developmental stages, while the *y*-axis represents normalized counts using variance stabilizing

transformation in DEseq2. In all panels, the points and error bars are the average values and *SE*, respectively, based on normalized counts of three RNA-seq replicates. The gene ID and the names of their putative orthologs are shown on the top of the expression pattern plots.

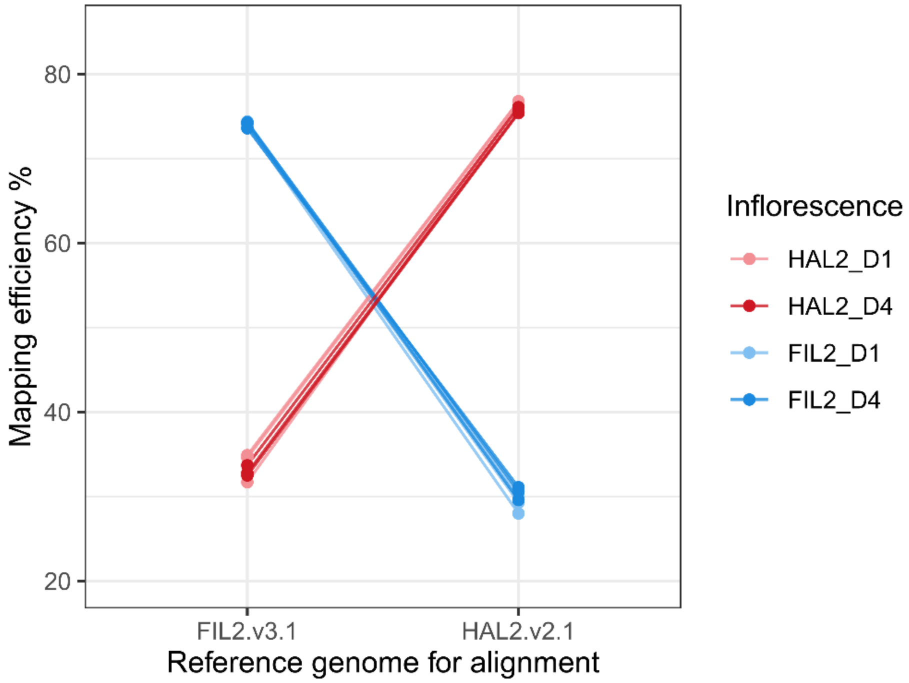

Supplemental Figure S7. Mapping efficiencies by performing alignments of all samples to both HAL2 or FIL2 reference genomes. The *x*-axis represents the references from FIL2 v3.1 and HAL2 v2.1, while the *y*-axis represents the mapping efficiency.

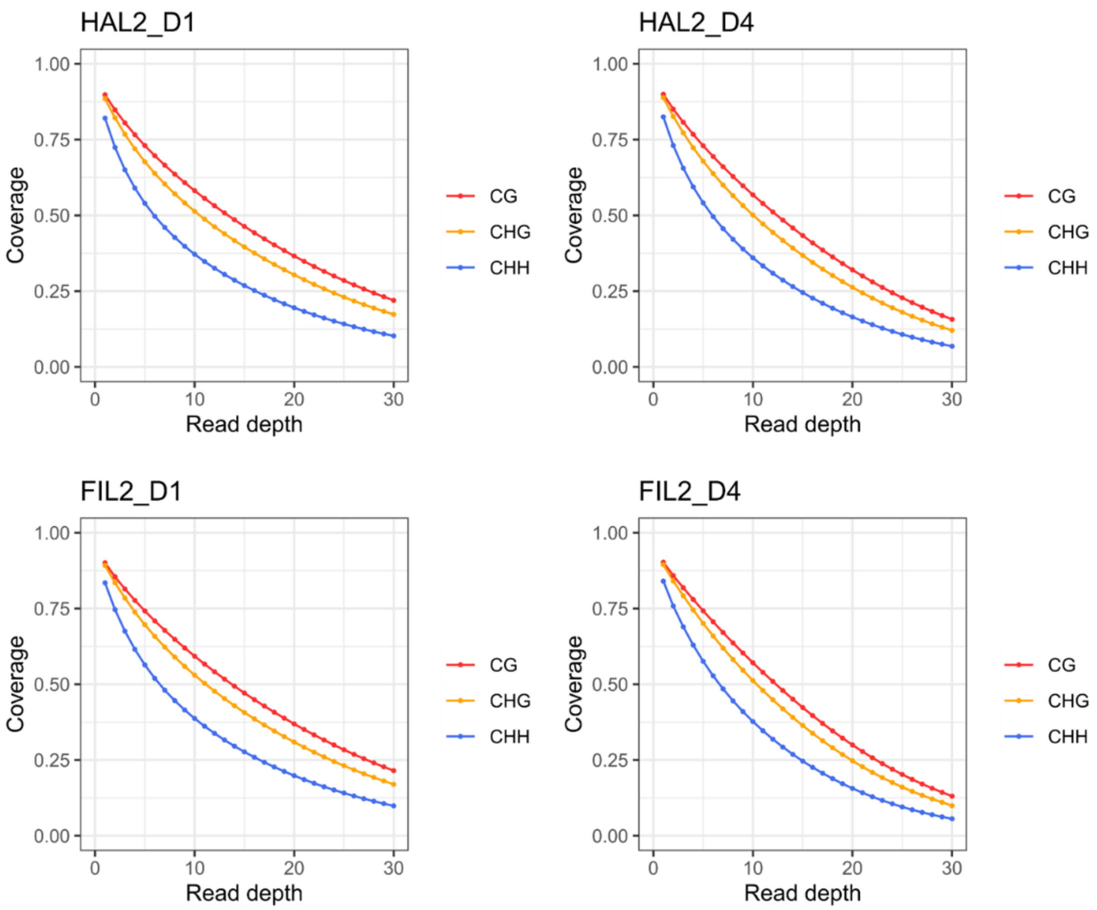

Supplemental Figure S8. Global read coverage distribution of cytosine in each context for all samples. Methylation coverage calculated as the proportion of cytosine positions in the HAL2 or FIL2 genome having at least a read depth from 1 to 30 reads, respectively (indicated in the *x*-axes). The average of three replicates was displayed for CG (red), CHG (yellow), and CHH (blue) contexts.

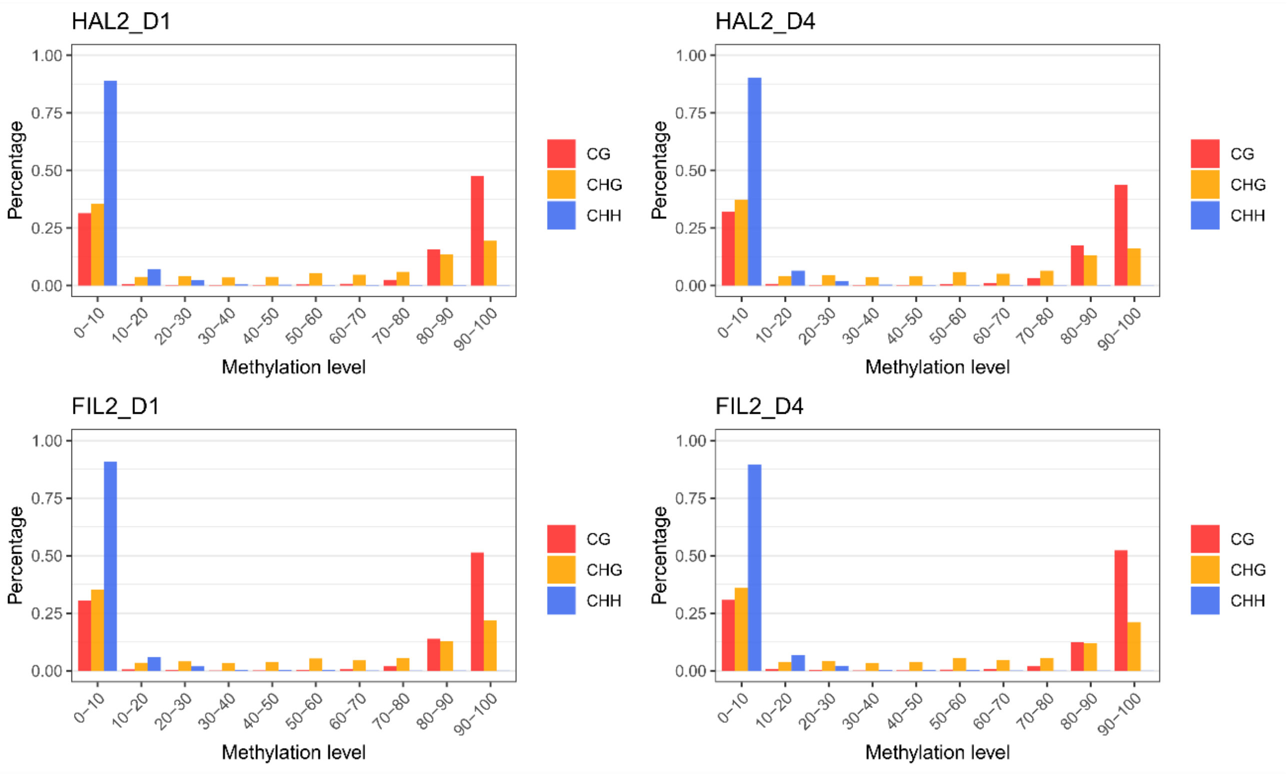

Supplemental Figure S9. Global distribution of methylation levels in each context for all samples. The *y*-axis indicates the frequency observed for the methylated cytosines that display the percentage of methylation indicated on the *x*-axis. Fractions were calculated within bins of 10%, as indicated on the *x*-axis. The average of three replicates was displayed for CG (red), CHG (yellow), and CHH (blue) contexts.

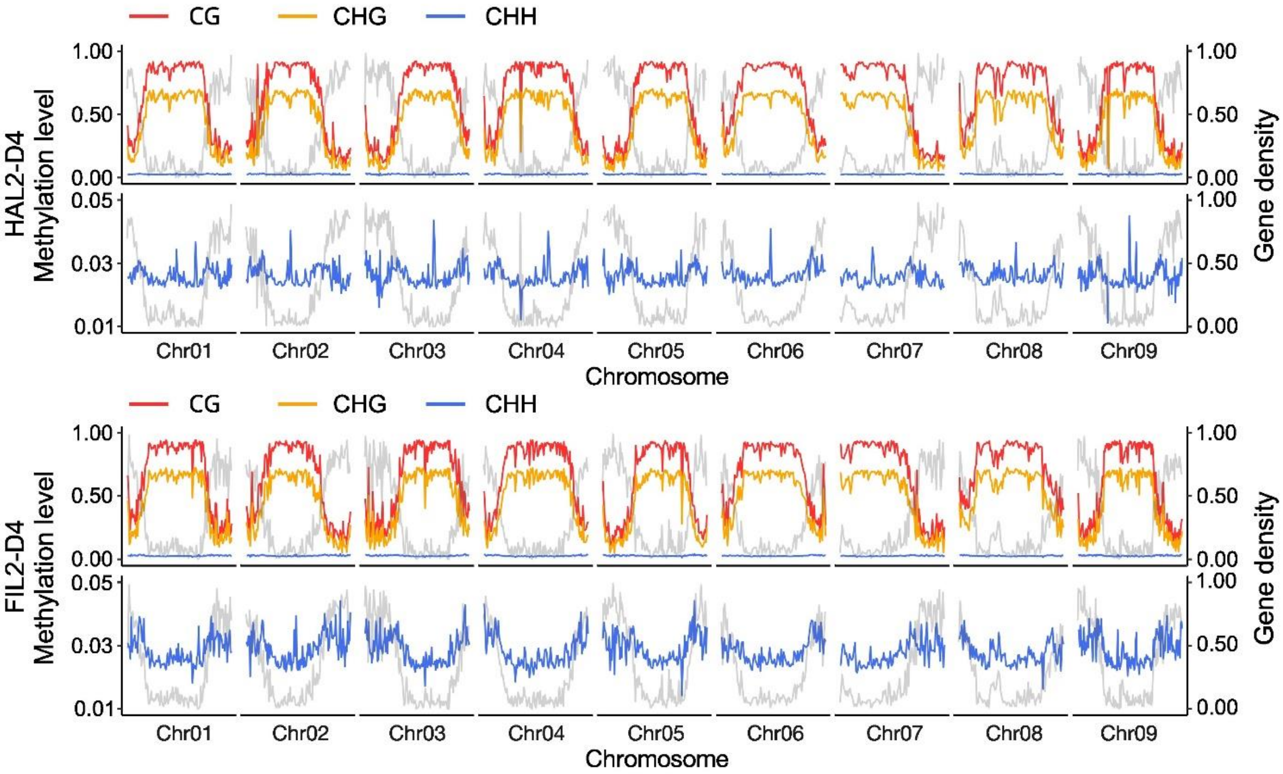

Supplemental Figure S10. Global DNA methylation profiling of HAL2 and FIL2 at the D4 stage of inflorescence development. The distribution of CG, CHG, and CHH methylation levels (mean values of three biological replicates) and gene density across chromosomes are presented in HAL2 and FIL2.

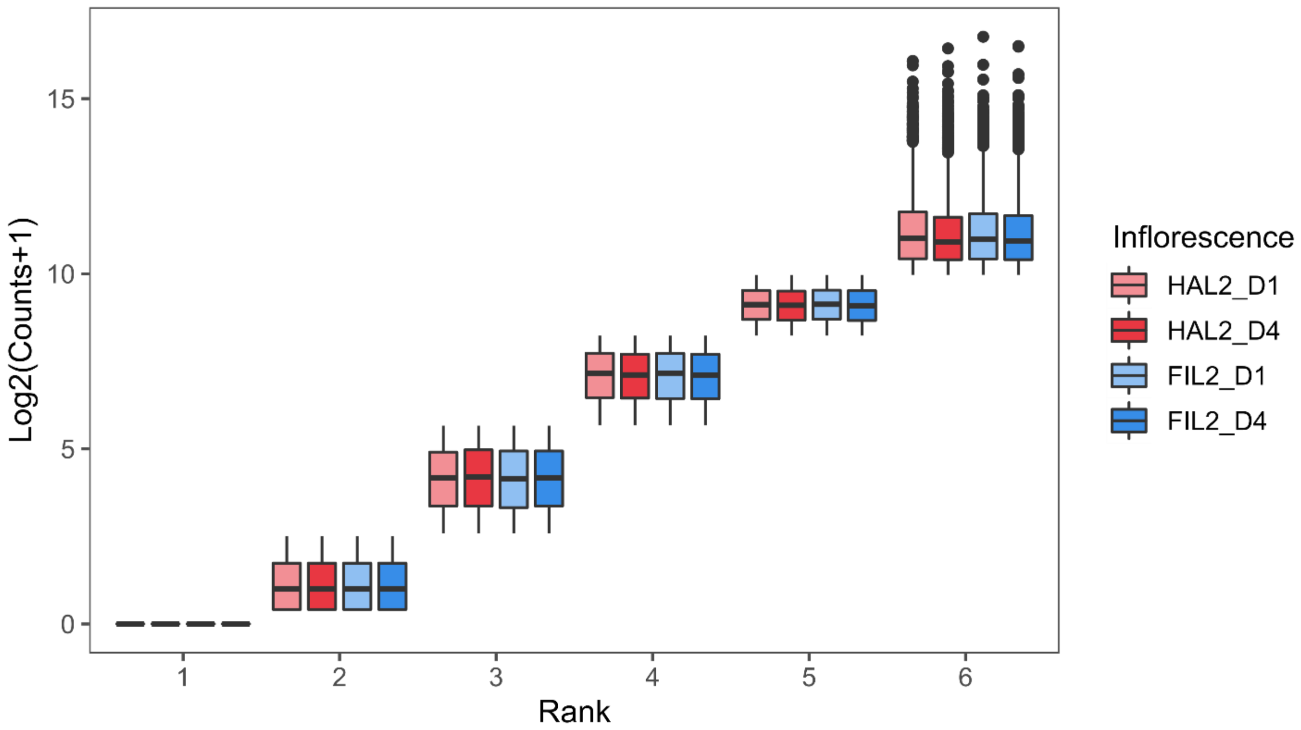

Supplemental Figure S11. The expression levels of six gene groups for comparison to methylation level across gene regions. All expressed genes were divided into six groups based on expression, from a silent rank1 (count = 0) to the highest rank6, as indicated on the *x*-axis. The *y*-axis represents the $\log_2$ transform expression level. The various elements of the boxplot were defined as below: center line refers to the median; box limits include upper and lower quartiles; whiskers refer to the 1.5 × interquartile range; points refer to outliers.

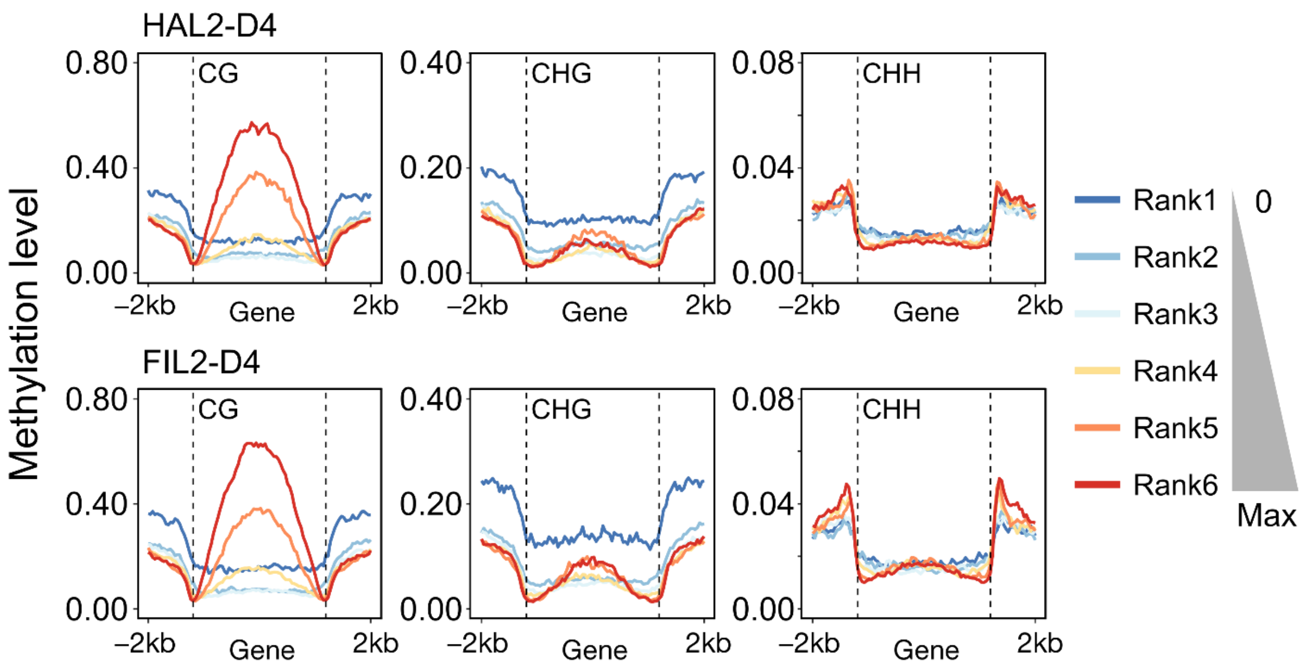

Supplemental Figure S12. Influence of DNA methylation on gene expression of HAL2 and FIL2 at the D4 stage of inflorescence development. Methylation level within gene body and 2 kb flanking regions in CG, CHG, and CHH contexts for the gene sets that are expressed at different levels in HAL2 and FIL2 D4 inflorescences. The average of three replicates was displayed for CG, CHG, and CHH contexts.

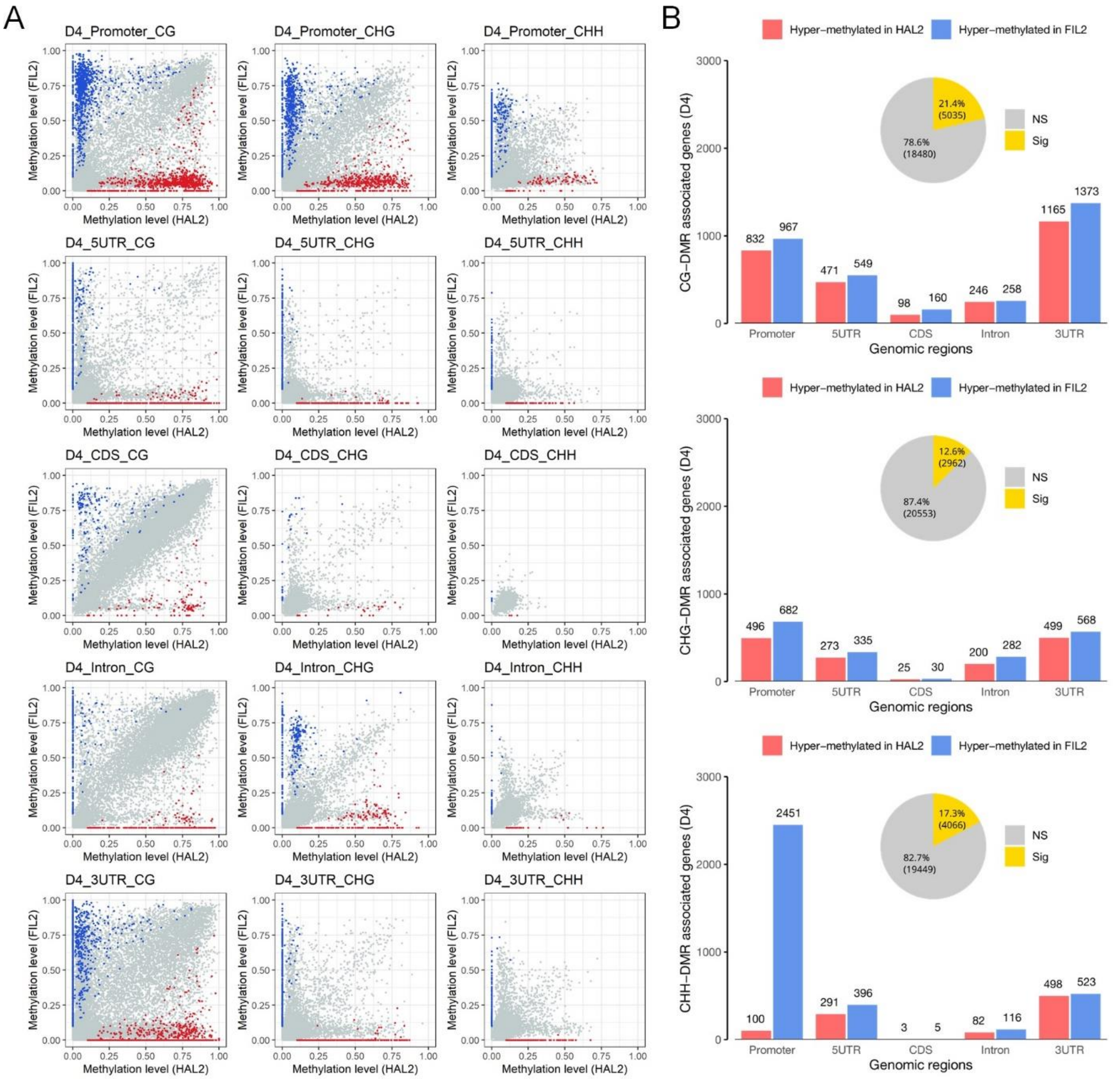

Supplemental Figure S13. Differential DNA methylation regions between HAL2 and FIL2 inflorescences at D4 stage of inflorescence development. (A) Pairwise comparisons of methylation levels from one-to-one orthologous gene pairs between HAL2 and FIL2 D4 inflorescence in CG, CHG, and CHH contexts across five different genomic features. Blue dots represent genes with significant hypermethylation in FIL2, while red dots represent genes with significant hypermethylation in HAL2. Grey dots represent genes with no significant methylation difference. (B) Number of differentially methylated genes between HAL2 and FIL2 D4 inflorescence in CG, CHG, and CHH contexts across

five different genomic features are shown in bar plots. The total number of differentially methylated genes in each context is shown in the associated pie chart. In the pie charts, “NS” refers to non-significant methylation difference, while “Sig” refers to significant methylation difference (A cut-off of < 0.01 *q*-value and > 0.1 methylation change were used to identify significant methylation difference).

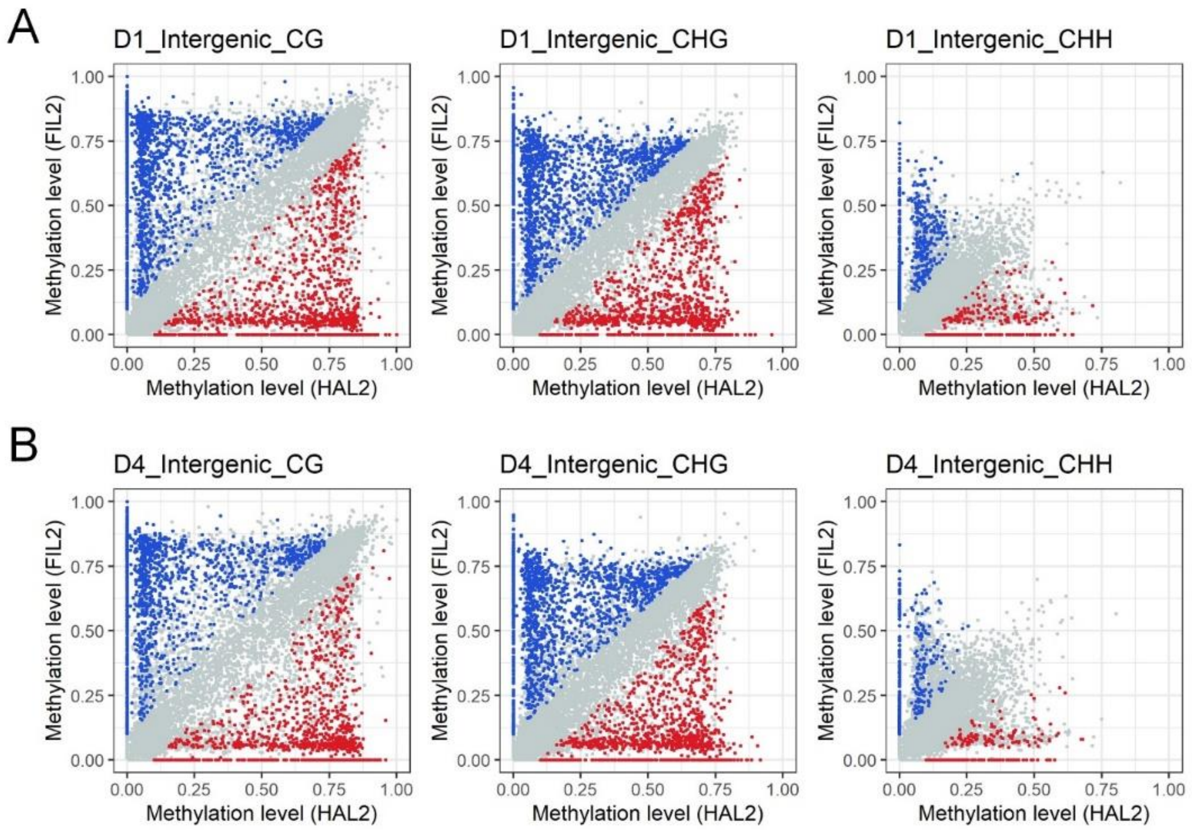

Supplemental Figure S14. Pairwise comparisons of methylation levels from paired intergenic regions between HAL2 and FIL2 inflorescence in CG, CHG, and CHH contexts at D1 (A) and D4 (B) stages. Blue dots represent genes with significant hypermethylation in FIL2, while red dots represent genes with significant hypermethylation in HAL2. Grey dots represent genes with no significant methylation difference.

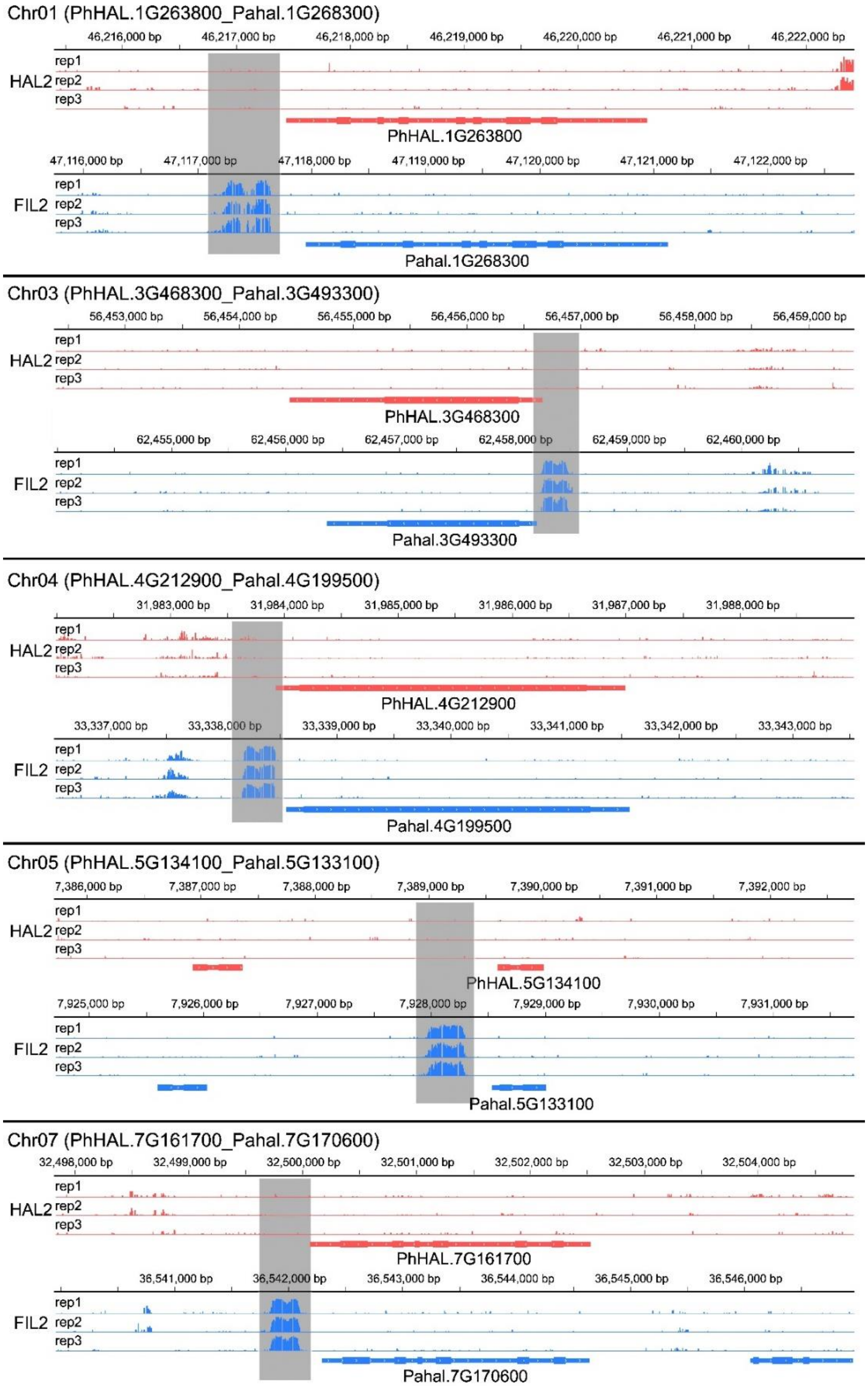

Supplemental Figure S15. Examples with considerable CHH hypermethylation (highlighted in grey boxes) in the promoter regions in FIL2 genes.

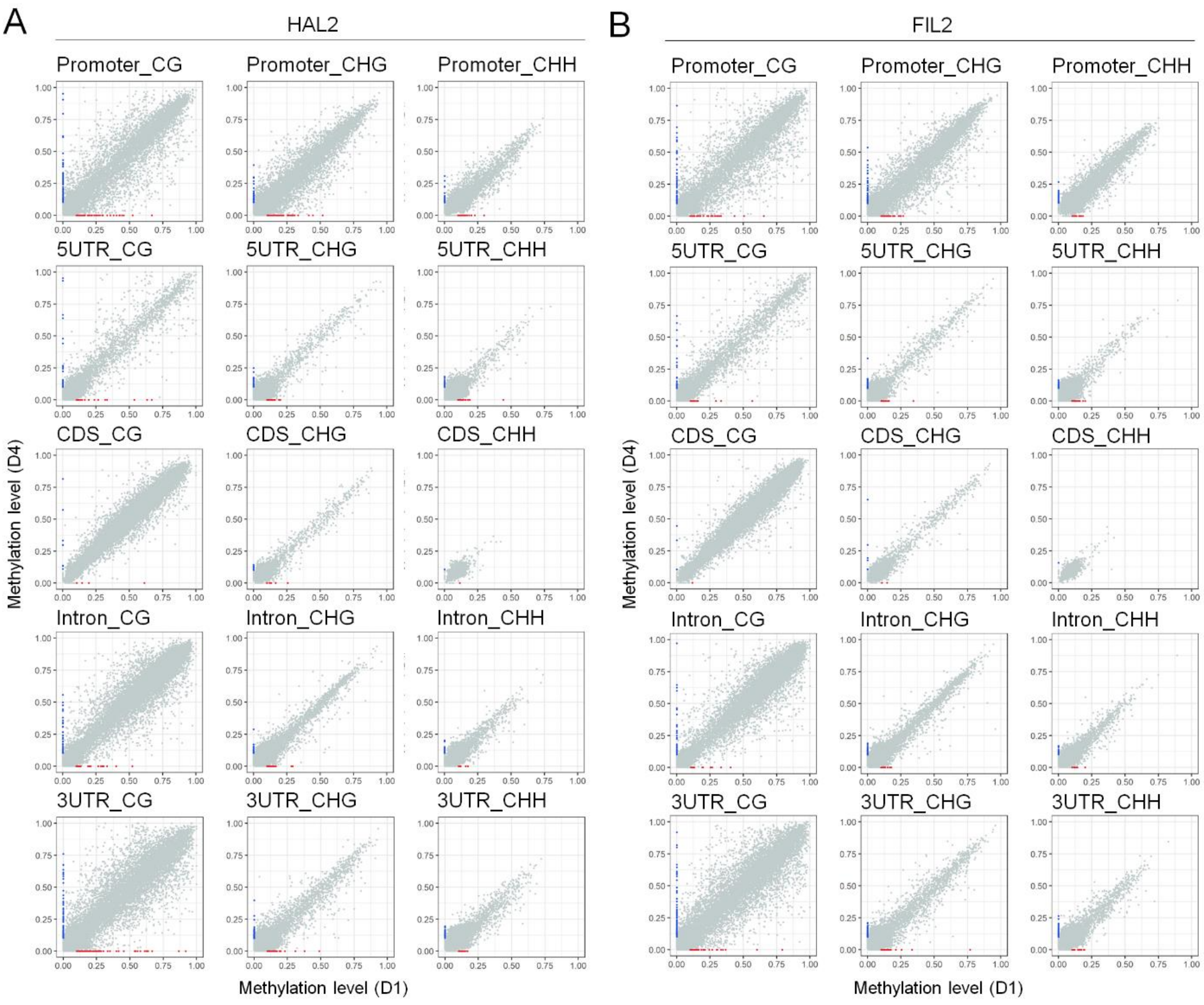

Supplemental Figure S16. Pairwise comparisons of methylation levels from one-to-one putative ortholog pairs between D1 and D4 in HAL2 (A) and FIL2 (B) inflorescence in CG, CHG, and CHH contexts across five different genomic features. Blue dots represent genes with significant hypermethylation in D4, while red dots represent genes with significant hypermethylation in D1. Grey dots represent genes with no significant methylation difference.

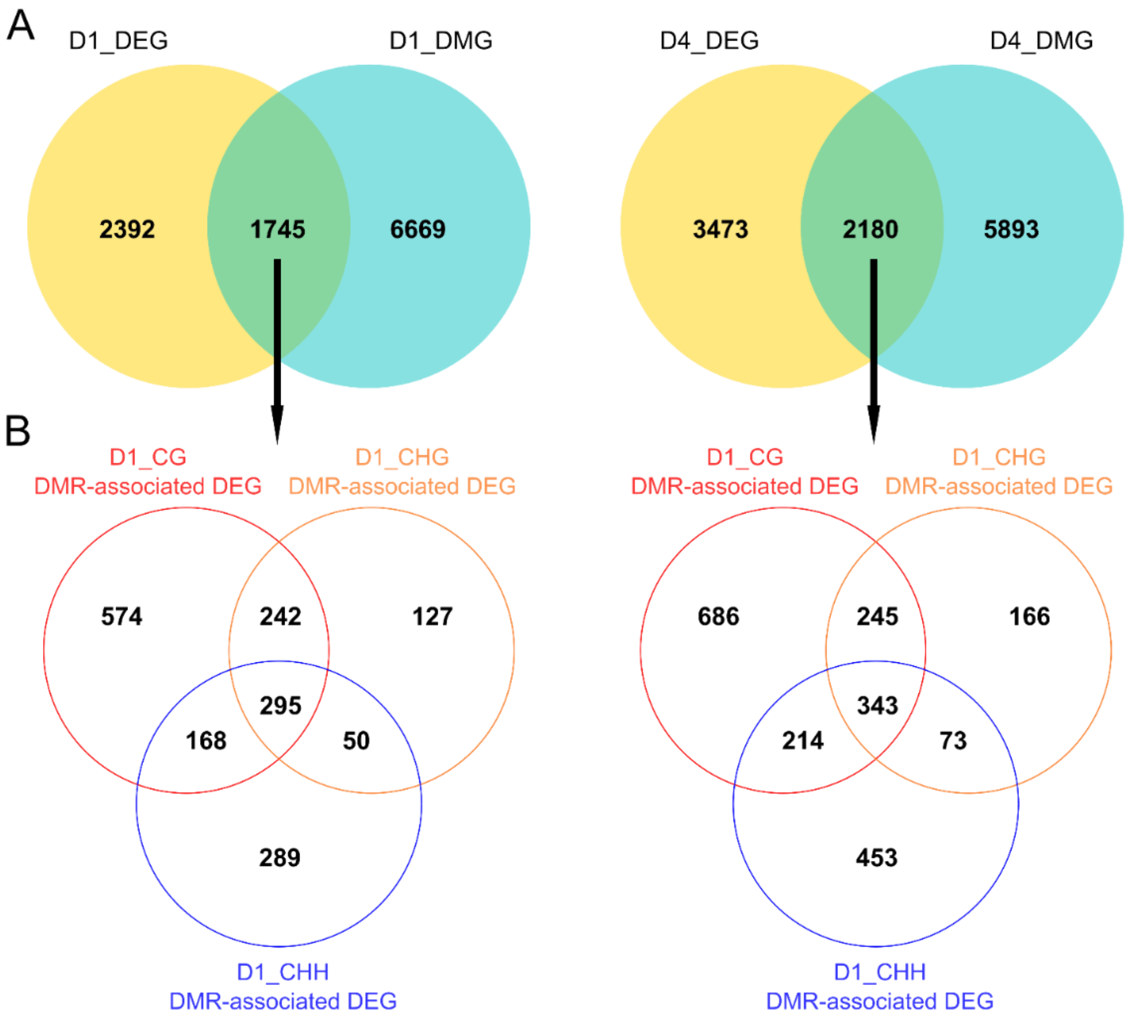

Supplemental Figure S17. Differentially methylation and expressed genes between HAL2 and FIL2 inflorescence. (A) Number of differentially methylated genes (yellow solid circle) and differentially expressed genes (cyan solid circle) between HAL2 and FIL2 inflorescence at D1 (left) and D4 (right) stages are given in Venn diagrams. (B) Number of differentially methylated regions (DMRs)-associated differentially expressed genes (DEGs) in CG, CHG, and CHH contexts between HAL2 and FIL2 inflorescence at D1 (left) and D4 (right) stages are given in Venn diagrams.

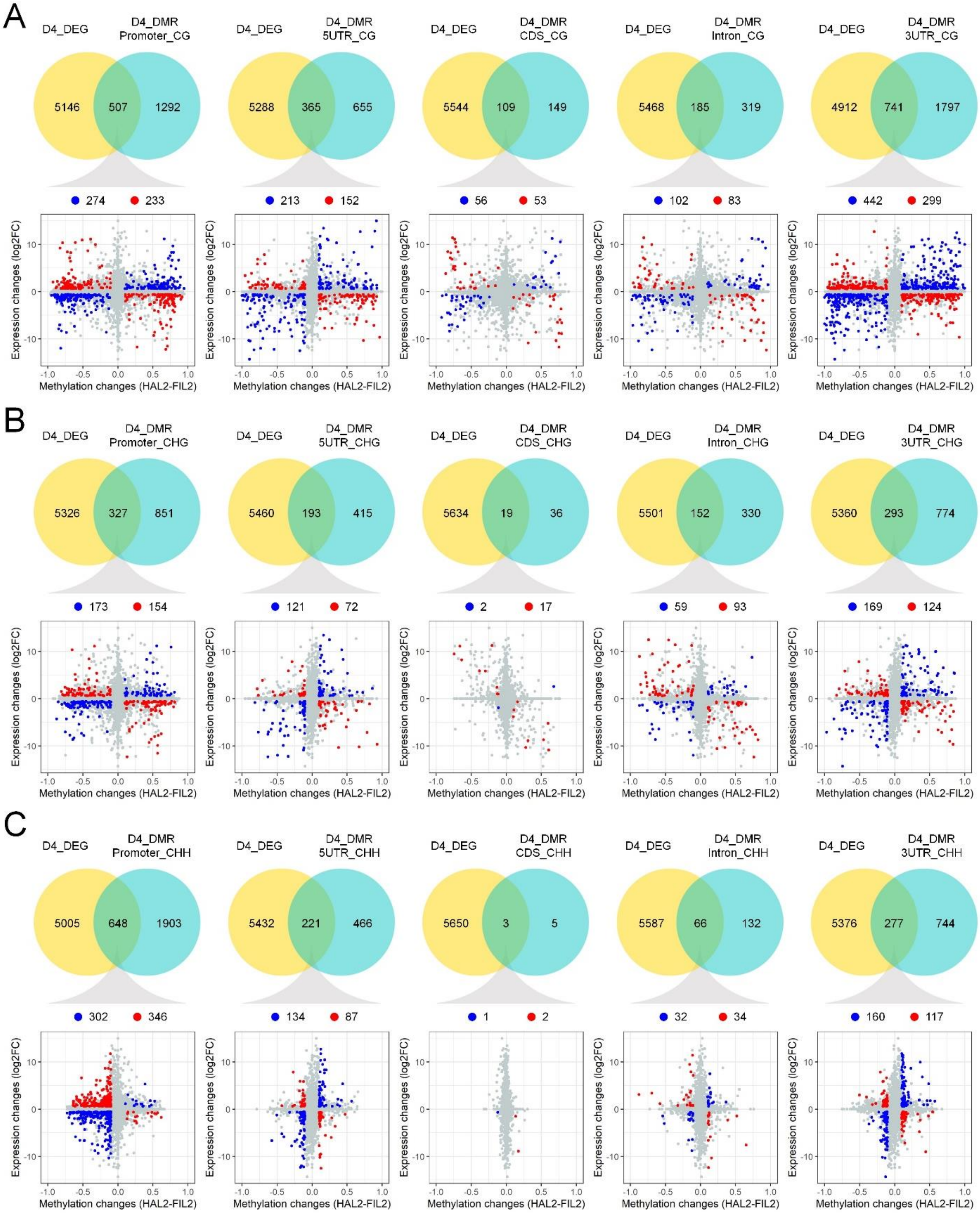

Supplemental Figure S18. Association of differentially methylated genes with differentially expressed genes at D4 stage. Venn diagrams depicting the number of differentially expressed genes (yellow circle,

DEGs) and differentially methylated regions (DMRs)-associated genes (blue circle, DMRs) between HAL2 and FIL2 D4 inflorescence in CG (A), CHG (B), and CHH (C) contexts across five different genomic features. Two-dimensional scatter plots depict the association of DEGs and DMRs in CG (A), CHG (B), and CHH (C) contexts across five different genomic features. The *x*-axis represents relative gene expression change (log2fold change), while the *y*-axis represents relative methylation change (HAL2 subtract FIL2).
